# The Sez6 Family Inhibits Complement by Facilitating Factor I Cleavage of C3b and Accelerating the Decay of C3 Convertases

**DOI:** 10.3389/fimmu.2021.607641

**Published:** 2021-04-15

**Authors:** Wen Q. Qiu, Shaopeiwen Luo, Stefanie A. Ma, Priyanka Saminathan, Herman Li, Jenny M. Gunnersen, Harris A. Gelbard, Jennetta W. Hammond

**Affiliations:** ^1^ Center for Neurotherapeutics Discovery, Department of Neurology, University of Rochester Medical Center, Rochester, NY, United States; ^2^ Department of Anatomy and Neuroscience and The Florey Institute of Neuroscience and Mental Health, University of Melbourne, Melbourne, VIC, Australia

**Keywords:** Sez6, Sez6L, Sez6L2, complement, classical pathway, alternative pathway, Factor I cofactor, C3 convertase

## Abstract

The Sez6 family consists of Sez6, Sez6L, and Sez6L2. Its members are expressed throughout the brain and have been shown to influence synapse numbers and dendritic morphology. They are also linked to various neurological and psychiatric disorders. All Sez6 family members contain 2-3 CUB domains and 5 complement control protein (CCP) domains, suggesting that they may be involved in complement regulation. We show that Sez6 family members inhibit C3b/iC3b opsonization by the classical and alternative pathways with varying degrees of efficacy. For the classical pathway, Sez6 is a strong inhibitor, Sez6L2 is a moderate inhibitor, and Sez6L is a weak inhibitor. For the alternative pathway, the complement inhibitory activity of Sez6, Sez6L, and Sez6L2 all equaled or exceeded the activity of the known complement regulator MCP. Using Sez6L2 as the representative family member, we show that it specifically accelerates the dissociation of C3 convertases. Sez6L2 also functions as a cofactor for Factor I to facilitate the cleavage of C3b; however, Sez6L2 has no cofactor activity toward C4b. In summary, the Sez6 family are novel complement regulators that inhibit C3 convertases and promote C3b degradation.

## Introduction

The Sez6 family, consisting of Sez6, Sez6L, and Sez6L2, is notable because its members have been identified as potential susceptibility genes for multiple neurodevelopmental and psychiatric disorders including: autism, schizophrenia, intellectual disability, epilepsy, and bipolar disorder (1–9). Sez6L2 is located in the 16p11.2 deletion/duplication region which encompasses 26 genes and accounts for ~1% of all autism spectrum disorder cases, but also has strong links to schizophrenia and intellectual disability (3, 4, 10). Sez6 family proteins are expressed by neurons throughout the brain during development and in adulthood. Some brain regions express multiple Sez6 family members, suggesting possible redundancy, while other regions have differential expression (11–15). In experimental mouse models, the Sez6 family has been shown to modulate synapse numbers, synaptic plasticity, and dendrite morphology in the cortex and hippocampus and neuronal connectivity in the cerebellum. Genetic loss of Sez6 genes results in impaired cognition, motor learning, and motor functions (13–16). Outside the nervous system, Sez6 family members have been implicated as markers of poor prognosis in cancer (17–23). Although a few binding partners of the Sez6 family have been proposed (24–28), the molecular mechanisms and functions of Sez6 proteins are still unclear.

The domain structure of Sez6 family members consisting of 5 complement control protein (CCP) domains and 2-3 CUB domains, suggest they may have activity connected to the complement cascade. CCP domains, also known as short consensus repeats or SUSHI repeats, contain 60-70 amino acids with four invariant cysteines forming disulfide bonds. CCP domains are the primary components of several proteins of the complement system (29, 30). However, CCP domains are also found in a variety of proteins outside the complement pathway involved in cell adhesion, blood coagulation, and signal transduction related to cytokines and neurotransmission (29–32). CUB domains are named after their founding members: complement C1r/C1s, uEGF, and BMP1 and primarily mediate protein-protein interactions that are often calcium-dependent. CUB domains are found in complement proteins such as C1s, C1r, MASP-1/2, and C3 as well as many other non-complement pathway proteins (33, 34). In addition to the CCP and CUB domains, Sez6 proteins have a single transmembrane domain and short cytoplasmic tail. The goal of this study was to evaluate the Sez6 family for complement regulatory functions.

The complement system has three initiating pathways (classical, alternative, and lectin) that all create C3 convertases to cleave C3 [reviewed in (35, 36)]. The classical/lectin pathway C3 convertase is composed of C4b2b [formerly called C4b2a (37)]. The alternative pathway convertase is composed of C3bBb. Proteolytic cleavage of C3 generates the chemotactic peptide C3a, as well as, C3b, which covalently attaches to activating surfaces such as antibody complexes, cellular debris, foreign particles, or even an organism’s own healthy “self” tissue. C3b and its degradation products iC3b and C3dg/d are opsonins that facilitate phagocytosis. When C3b is sufficiently accumulated, it associates with C3 convertases to form C5 convertases. C5 convertases cleave C5 to initiate the lytic complement pathway leading to cell lysis.

Complement activation on “self” surfaces is controlled by a group of complement regulatory proteins. A common feature of complement regulators that inhibit C3 convertases is the presence of three or more tandem CCP domains (29, 38). The most well-known CCP containing complement regulators are DAF/CD55, MCP/CD46, and CR1 (or Crry in rodents) which are membrane-associated and Factor H (FH) and C4BP, which are soluble circulating factors (35, 36). Complement regulators can deactivate C3 convertases by dissociating the protease subunits (Bb/2b) from the non-catalytic subunits (C3b/C4b), which has been defined as “decay accelerating” activity. Complement regulators can also facilitate cleavage of C3b or C4b by functioning as cofactors for the protease, complement Factor I (29, 36, 38). Cleaved C3b fragments (iC3b, C3c, C3dg/d) or C4b fragments can no longer function as subunits for C3 or C5 convertases.

The complement system is a fundamental part of the innate immune system, but it also has important roles in neural development. These roles include neurogenesis, synapse pruning, and neuronal migration (39–44). The complement pathway additionally mediates immune responses to prenatal or early postnatal brain insults that affect brain development with lifelong consequences [reviewed in (44)]. Furthermore, complement contributes to pathological cell and synapse loss in aging and diseases including Alzheimer’s, viral encephalitis, glaucoma, lupus, epilepsy, schizophrenia, frontotemporal dementia, and multiple sclerosis (39–41, 45–58). Therefore, understanding complement regulation in the brain is important for neurodevelopment as well as neurodegenerative disease. We show here that members of the Sez6 family are novel complement regulators that inhibit the complement pathway at the level of C3 convertases.

## Materials and Methods

### Purified Proteins

Cloning and Plasmids: The expression plasmid for Sez6L2-MH, a truncated version of human Sez6L2 that has the transmembrane and cytoplasmic region replaced by a tandem Myc/6x-His tag, was cloned using PCR from the Sez6L2 NCBI Refseq BC000567 cDNA (similar to NM_001114100.2; Uniprot ACC: Q6UXD5-6); and the following primers: 5’–GACTCGAGAATTCGCGGCCGCCACCATGGGGACTCCCAGGGCCCAGCA-3’ and 5’-TTCTAGAAAGCTTGGTACCTCCCCCCCTTCCAGCTGCCGTGATGG-3’. The PCR product was digested with restriction enzymes Not1 and HindIII and cloned into the mammalian expression plasmid, pEZYmyc-His [Addgene plasmid #18701; http://n2t.net/addgene:18702; RRID: Addgene_18701; a gift from Yu-Zhu Zhang (59)], that was digested with the same enzymes. The human His-DAF plasmid from Sino Biological (HG10101-NH; modified from NCBI RefSeq NM_000574.3) was used for protein production of H-DAF.

His-Tagged Protein Purification (Sez6L2-MH and H-DAF): FreeStyle 293-F cells grown in serum-free FreeStyle 293 Expression Medium (Gibco, 12338-018) were transfected with Sez6L2-MH or His-DAF plasmids using Fectopro (Polyplus, 116-001). After 48-72 hours, media was collected and His-tagged proteins were bound using HisPur Ni-NTA Resin (ThermoFisher; 88221) in the presence of Calbiochem’s EDTA-free protease inhibitor cocktail 1:1000 (MilliporeSigma; Set V; 539137). Bound proteins were eluted in 250 mM imidazole in PBS pH 7.4 and filtered through a 0.22 µm membrane (such as MilliporeSigma, SCGP00525). Elutions were then either dialyzed with PBS overnight (MWCO 12-14K, Spectrum Spectra/Por Molecular porous tubing, 132706) and concentrated using Amicon Ultra filters MWCO-10K or 30K (MilliporeSigma; 10K: UFC901024, 30K: UFC903024) or directly concentrated and subjected to multiple rounds of buffer exchange to PBS using the Amicon Ultra filters or a similar product (Pierce 30K MWCO concentrator, 88529). If protein concentration resulted in visible protein aggregates, the solution was again filtered through a 0.22 µm membrane (such as MilliporeSigma Millex-GV4 SLGVL0405). Protein concentration was determined by absorbance at 280 nm with a 1% extinction coefficient correction (E1%) of 11.4 for Sez6L2-MH and 10.6 for H-DAF. The 1% extinction coefficients were calculated based off the amino acid sequence using ProtParam (http://www.protparam.net/index.html). Protein aliquots were frozen and stored at -80°C.

Other Purified Proteins: The negative control protein, bovine serum albumin (BSA), was purchased from MilliporeSigma (A3294). In order to make the BSA solution as similar to Sez6L2-MH and H-DAF as possible, we processed BSA through the last steps of the His-tagged protein purification protocol. Specifically, we dissolved the lyophilized BSA in PBS with 250 mM imidazole (the same buffer used to eluted proteins from HisPur Ni-NTA Resin). The BSA solution was then concentrated, exchanged to plain PBS, and stored at -80°C. All other purified proteins were purchased from CompTech: C1s-enzyme (A104), C2 (A112), C3 (A113); C3b (A114), iC3b (A115), C3d (A117), C4 (A105), C4b (A108), C4bBP (A109), Factor B (A135), Factor D (A136), Factor H (A137), Factor I (A138). These proteins were purified from human serum and were in PBS.

### Hemolytic Assay

Classical pathway: Complement mediated lysis of erythrocytes releases hemoglobin that can be measured by absorbance spectrophotometry at 415 nm. Antibody-sensitized sheep erythrocytes (EA; CompTech; B200) were rinsed and diluted in GVB^++^ buffer (0.1% gelatin, 5 mM Veronal, 145 mM NaCl, 0.15 mM CaCl_2_ 0.5 mM MgCl_2_. 0.025% NaN_3,_ pH 7.3). 5 µM of Sez6L2-MH or other purified proteins were pre-incubated in a v-bottom 96 well plate for 15 minutes on ice with 0.275% normal human serum (NHS, CompTech) in a final 90 µl buffer solution equivalent to 40% GVB^++^ and 50% PBS with 0.15 mM CaCl_2_ and 0.5 mM MgCl_2_ (PBS^++^). Next, 10 µl of EAs (4x10^8^ cells/mL in GVB^++^) were added to each sample and the plate was incubated at 37⁰C for 30 minutes. During this incubation, cells were suspended with a multichannel pipet every 10 minutes. The reaction was stopped by adding 100 µl of cold GVBE buffer (0.1% gelatin, 5 mM Veronal, 145 mM NaCl, 0.025% NaN_3_, 10 mM EDTA, pH 7.3). Remaining erythrocytes were pelleted by centrifugation at 500xg for 3 minutes. 150 µl of the supernatant was transferred to a flat-bottom 96-well plate and measured for absorbance at 415 nm using a microtiter plate reader (SpectraMax M5). The absorbance obtained with just buffer, NHS, and EAs was set at 100% lysis and the absorbance obtained without serum added or when GVBE was used in place of GVB^++^ to inhibit complement was set at 0% lysis. Absorbance from full lysis in each experiment was also determined by replacing PBS with H_2_0 (at 50% final sample volume). Prior to testing the efficacy of complement inhibitors, the amount of each lot of normal human serum necessary to lyse EAs to 80-90% of the absorbance obtained by H_2_0 samples was determined by testing a range of NHS concentrations from 0-2%. These titrations were done to ensure that inhibitors were not overwhelmed by saturating levels of complement activity. The titrations revealed that 80-90% lysis was usually obtained with 0.25-0.4% serum.

Alternative Pathway: The alternative pathway hemolytic assays were performed similar to the classical pathway assay except that plain rabbit erythrocytes (Er, CompTech) were rinsed and diluted in GVB° (0.1% gelatin, 5 mM Veronal, 145 mM NaCl, 0.025% NaN_3_, pH 7.3). 5 µM Sez6L2-MH or other purified proteins were pre-incubated for 15 minutes on ice with 6.5-7% normal human serum in a final 95 µl buffer solution containing 10 mM MgCl_2_ and 10 mM EGTA and the equivalence of 45% GVB° and 50% PBS. Next, 5 µl of Ers (5x10^8^ cells/mL in GVB°) were added to each sample and the plate was incubated at 37°C for 30 minutes and processed similar to the classical hemolysis experiments. The 415 nm absorbance obtained with just buffer, NHS, and Ers was set at 100% lysis.

### Cleavage of C3b or C4b by Factor I

C3b (1.5 µM; 3.94 µg) or C4b (1.5 µM; 4.05 µg) was incubated with 0.5 µM Factor I alone or in the presence of 1 µM FH, 1 µM C4BP, or 1-8 µM Sez6L2 in PBS^++^ (Total volume ~15 µl) for 15 minutes-8 hours at 37°C. The samples were then run on reducing SDS-PAGE gels and analyzed by western blot or Imperial Blue (Coomassie) staining to identify C3 or C4 cleavage products.

### Decay Accelerating ELISAs

Alternative C3 Convertase Decay Assay: An ELISA-based assay was used to measure decay accelerating activity towards the alternative pathway C3 convertase, C3bBb. ELISA plates (Nunc, MaxiSorp, 96 well plates, 44-2404-21) were coated with 100 µl human C3b at 3-3.5 µg/mL (17-20 nM) in PBS for ~20 hours at room temperature. Wells were washed twice with PBS and blocked with 1% BSA in PBS for 30-60 minutes at room temperature. Wells were again washed twice with PBS. C3 convertase was formed by incubating with Factor B (5.8-10 nM) and Factor D (1.8-2.0 nM) in GVB° buffer with 3.5 mM NiCl_2_ at 37°C for 30 minutes. Subsequently, wells were washed three times with PBSt (0.1% tween). Factor H and Sez6L2-MH were mixed at various concentrations ranging from 0-6 µM (0 µg/mL to 500 µg/mL) in solutions with a final composition of 90 µL PBS to 225 µL GVB° with 3.5 mM NiCl_2_. 100 µl of the Factor H or Sez6L2-MH mixtures were added to the plate in triplicate and incubated at 37°C for 45 minutes. Supernatants were collected for western blot analysis and the wells were washed three times with PBSt. Residual Bb on the plate was detected using polyclonal goat anti-Factor B (CompTech, A235, 1:4,000) for one hour at room temperature. Wells were washed three times with PBSt and incubated with a donkey HRP-conjugated anti-Goat IgG (Azure Biosystems, AC2149, 1:10,000) for 45 minutes at room temperature. Wells were then washed three times with PBSt prior to colorimetric development using TMB substrate (ThermoScientific, N301). 0.16 M sulfuric acid was used to stop the reaction. Absorbance was measured at 450 nm. Percent convertase remaining was calculated with the following formula = ((Ab450 inhibitor – Ab450 background)/(A450 buffer – Ab450 background)) x 100. Background values were obtained from wells not coated with C3b but incubated with Factor B and D. Nonlinear regression utilized a variable slope and four parameters.

Classical/Lectin C3 Convertase Decay Assay: This ELISA-based assay was performed similar to the C3bBb assay outlined above but with the following changes: Plates were coated with 3 µg/mL (16.7 nM) C4b in PBS. After washing and blocking with 1% BSA, C3 convertases were formed by incubating with C2 (6-12 nM) and C1s-enzyme (4 nM) in GVB++ at 37°C for 30-60 minutes. After, wells were washed three times with PBS^++^. H-DAF and Sez6L2-MH were mixed at various concentrations ranging from 0-7 µM in solutions with a final composition of 50% GVB^++^ and 50% PBS^++^. 100 µl of the H-DAF or Sez6L2-MH mixtures were added to the plate in triplicate and incubated at 37°C for 30 minutes. Wells were washed three times with PBSt^++^ (containing 0.1% tween). Residual C2b/C2 on the plate was detected using polyclonal goat anti-C2 (CompTech, A212, 1:6,000 in PBSt^++^) for 45 minutes at room temperature. Wells were washed three times with PBSt^++^ and then incubated with a donkey HRP-conjugated anti-Goat IgG (Azure Biosystems, AC2149 1:10,000 in PBSt^++^) for 45 minutes at room temperature and then processed as above. Background values were obtained from wells not coated with C4b but incubated with C2 and C1s-enzyme.

### Flow Cytometry

CHO cells (a Chinese hamster ovary cell line) were grown in serum-free Freestyle media (Gibco, 12651-014) and transfected with human cDNA expression plasmids with N-terminal Myc-tags obtained from Sino Biological: M-SEZ6L2 (HG13969-NM; modified from NCBI RefSeq BC000567, similar to NM_001114100.2; Uniprot ACC: Q6UXD5-6), M-SEZ6 (HG13436-NM; modified from NCBI RefSeq NM_178860.4; Uniprot ACC: Q53EL9-1), M-SEZ6L (HG20982-NM; modified from NM_001184773.1; Uniprot ACC: Q9BYH1-6), M-MCP (HG12239-NM; modified from NCBI RefSeq BC030594), or M-CR2 (HG10811-NM; modified from NCBI RefSeq NM_001877.3) using FectoPro transfection reagent (Polyplus, 116-001). Alternatively, cells were transfected with a GFP expression plasmid or co-transfected with GFP and M-SEZ6L2 or HIS-tagged DAF (H-DAF; Sino Biological HG10101-NH; modified from NCBI RefSeq NM_000574.3). After 18-24 hours, the cells were collected and loaded at 4x10^5^ cells per well in a 96-well v-bottom plate. To assay the activity of the classical complement pathway, CHO cells were sensitized with a 30 minute incubation with rabbit anti-hamster lymphocyte serum (1:10; Cedarlane, CLA14940) in 100 µl cold FACs buffer (0.5% BSA in PBS) at 37°C. Cells were washed twice with FACs buffer then incubated with 15% C5-depleted human serum (CompTech, A320) or a range of C5-depleted serum from 0-20% in GVB++ at 37°C for 60 minutes. After washing twice with cold FACs buffer, cells were incubated with a mouse anti-human C3b/iC3b APC conjugated antibody (1:50; Biolegend, 846106, clone 3E7) and mouse anti-Myc Alexa488 conjugated antibody (1:50; Cell Signaling Technologies, 2279S, clone 9B11) for 30 minutes at 4°C. For samples transfected with GFP, only the anti-C3 APC antibody was used. After two washes in FACs buffer, cells were resuspended in FACs buffer with 1 µl propidium iodide (PI, Invitrogen, BMS500PI) and processed using a BD Accuri C6 Flow Cytometer. Data was analyzed by FlowJo™ Software (Windows Version 10.6.1: Ashland, OR: Becton, Dickinson and Company; 2019). Data were gated for the single cell population (FSC-A:FSC-H) and PI negative population. To assay the alternative pathway, cells were processed as above except they were sensitized with only a low level of anti-hamster lymphocyte serum (1:50) and the 20% C5-depleted serum was diluted in GVB° with 10 mM EGTA and 10 mM MgCl_2_ to block the classical pathway.

### Western Blot

Supernatants from decay accelerating ELISA’s or Factor I cleavage assays were mixed with SDS-loading dye and run on 8-15% SDS-PAGE gels and transferred to PVDF. Membranes were blocked with 5% milk in TBSt (Tris buffered saline (20 mM Tris-Cl, pH 7.4; 150 mM NaCl) with 0.1% Tween 20) for 30 minutes and probed with primary antibodies: polyclonal goat anti-C3d (R&D systems, AF2655, 1:1000), polyclonal sheep anti-Sez6L2 (R&D Systems, AF4916; 1:2000), monoclonal mouse anti-Myc (Developmental Studies Hybridoma Bank; 9E10, 1:2000), polyclonal goat anti-Factor B (CompTech, A235, 1:1000) in 5% milk in TBSt for one hour at room temperature or overnight at 4°C. Membranes were washed three times in TBSt and then incubated with HRP secondary antibodies (anti-Mouse HRP, BioRad, 170-6516, 1:5,000; anti-Goat HRP Azure Biosystems, AC2149, 1:10,000; anti-Sheep HRP, Jackson Labs, 713-035-003, 1:1000) for one hour at room temperature in 5% milk in TBSt. After washing we applied ECL substrate (Pierce) and developed membranes using a digital imager (Azure Biosystems). Some membranes were stripped using buffer consisting of 200 mM glycine; 0.1% SDS, 1% Tween 20, pH 2.2 and re-probed. Western blot band densities were quantitated using Image J/FIJI.

### Immunohistochemistry (IHC) and Imaging

Sez6 Triple Knockout mice (Sez6 TKO) have a complete knockout of Sez6, Sez6L and Sez6L2 (14, 15). They are also known as BSRP TKO mice and were supplied by co-author Dr. Jenny Gunnersen, University of Melbourne; Australia. Mice were anesthetized with ketamine/xylazine (100 and 10 mg/kg, respectively) and intracardially perfused with phosphate-buffered saline (PBS) containing EDTA (1.5 mg/ml) followed by 4% paraformaldehyde (PFA) in PBS. Brains were post-fixed for 18-30 hours in 4% PFA, then stored in PBS at 4°C. Brains were cut into 40 μm-thick coronal sections using a vibratome (Leica V1000) and stored in a cryoprotectant mixture of 30% PEG300, 30% glycerol, 20% 0.1 M phosphate buffer, and 20% ddH_2_O at −20°C. IHC was performed on free-floating sections. The sections were washed three times for 30 minutes in PBS to remove the cryoprotectant. Then sections were incubated in 100 mM glycine in PBS for 30 minutes followed by citrate antigen unmasking at 37°C for 30 minutes (Vector, H3300 with 0.05% tween 20). Sections were blocked overnight in blocking buffer (consisting of 1.5% BSA (MilliporeSigma; A3294), 3% normal donkey serum (Jackson Immunoresearch Laboratories, 017-000-121), 0.5% Triton X-100 (Promega, H5142), and 1.8% NaCl in PBS) with donkey anti-mouse Fab fragments (Jackson ImmunoResearch Laboratories, 715-007-003; 1:300). Sections were then washed three times in PBS with 1.8% NaCl. Primary antibodies: polyclonal sheep anti-Sez6L2 (R&D Systems, AF4916,1:500) and polyclonal chicken anti-Homer1 (Synaptic Systems, 160006, 1:500) were diluted in blocking buffer and incubated with brain sections for 1-3 days at room temperature with agitation. Sections were then washed three times for 30 minutes in 1× PBS with 1.8% NaCl and then incubated overnight at room temperature in Alexa Fluor-conjugated secondary antibodies (1:500 Jackson ImmunoResearch Laboratories Alexa 488 Donkey anti-sheep, Cat# 713-545-147, RRID: AB_2340745; and Alexa 647 Donkey anti-chicken, Cat# 703-605-155, RRID: AB_2340379) in blocking buffer. Finally, sections were washed three times with PBS with 1.8% NaCl, mounted on slides with Prolong Diamond mounting agents with DAPI (Life Technologies; P36962).

IHC sections were imaged with a Hamamatsu ORCA-ER camera on an Olympus BX-51 upright microscope with Quioptic Optigrid optical sectioning hardware with a 10x, NA 0.4 or 60x, NA 1.4 objective with *z*-step = 0.3 μm. Volocity 3DM software (Quorum Technologies; version 6.3) was used for image collection and analysis. Differences in *z*-axis registration of various fluors were corrected by calibration with multicolor fluorescent beads. Image stacks are displayed as maximum intensity protections.

### Statistics

GraphPad Prism software version 8.3.0 for Windows (La Jolla California USA) was used to perform all statistics. Statistical analysis was generally performed with one-way ANOVAs followed by Holm-Sidak’s multiple comparisons test (MCT). For the CHO cell C3b/iC3b deposition assays, Myc-positive and Myc-negative populations within the same sample were first compared with multiple t tests. Then, p values were corrected with Holm’s-Sidak’s MCT for the many samples within the experiment. N values for each experiment are specifically stated and defined in the figure legends. We defined significance as *p <*0.05 and used the following markings on graphs * p<0.05; ** p<0.01. All data are expressed as the mean ± standard error of the mean (SEM).

## Results

### Expression of Sez6 Family Proteins and Other Complement Regulatory Proteins in Hippocampal Neurons

**Figure 1:**
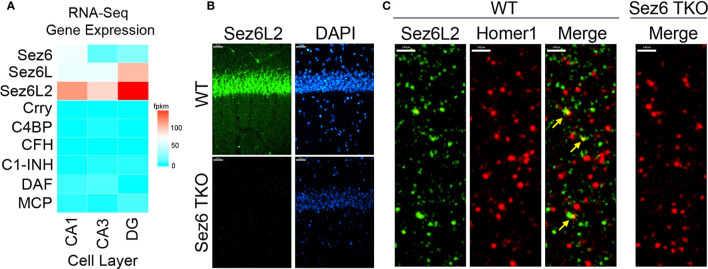
Sez6 family expression in the hippocampus. **(A)** Sez6, Sez6L, and Sez6L2 are expressed by principal (excitatory, pyramidal) neurons of the mouse hippocampus at much higher levels than other known complement regulators (namely Crry, C4BP, CFH, C1-INH, DAF, and MCP). Expression data was obtained from Hipposeq: a comprehensive RNA-Seq database of gene expression in hippocampal principal neurons [http://hipposeq.janelia.org (60)]. The RNA samples used in this database were isolated from mouse hippocampal principal neurons micro-dissected from the CA1, CA3, or Dentate Gyrus (DG) cell layers of the hippocampus at Postnatal Day 25-32. Differential gene expression is shown in the heatmap with the relative units of FPKM (Fragments per Kilobase of Exon per Million Reads Mapped.) **(B)** Brain sections from adult WT mice or Sez6 triple knockout mice (TKO) were immuno-stained for Sez6L2 (green) and DAPI and imaged in the CA1 region of the hippocampus. Scale Bar= 27 µm. High density Sez6L2 staining occurs around cell bodies in the pyramidal layer, but significant Sez6L2 is also found in the stratum radiatum and stratum oriens. **(C)** Higher magnification images of sections immuno-stained for Sez6L2 (green) and the postsynaptic protein, Homer1 (red), shows a subset of Sez6L2 is found near or co-localized with synapses in the stratum radiatum. Scale bar = 1.8 µm.

Self-directed complement activity is usually tightly controlled by complement regulatory proteins expressed on cell membranes and/or secreted in soluble form. However, the identity of complement regulators functioning on neurons throughout neuronal development or in disease with a potential role for modulating complement-mediated synaptic pruning has yet to be fully elucidated. Because hippocampal neurons express only trace amounts of the most well-known complement regulators of the early complement pathways (DAF, CR1/Crry, FH, C4BP, MCP or C1-INH (60) (**Figure 1A**), we searched the smart protein database (smart.embl-heidelberg.de/) for proteins with at least three tandem CCP domains that are also highly expressed by neurons in development. We identified the Sez6 family of proteins (consisting of Sez6, Sez6L, and Sez6L2) as ideal candidates. RNA-Seq expression data from the Hipposeq database [http://hipposeq.janelia.org (60)] show that the Sez6 family members are expressed by pyramidal neurons in the hippocampus with differential expression in CA1, CA3, and the dentate gyrus (**Figure 1A**). Immunostaining of adult mouse brain sections shows the Sez6L2 is highly localized to neuronal cell bodies of the CA1 region. Diffuse Sez6L2 is also found throughout the neuropil of the stratum radiatum and stratum oriens where a subset of Sez6L2 puncta co-localize with synapses (identified as Homer1+ puncta) (**Figures 1B, C**).

### Sez6 Proteins Limit C3b/iC3b Opsonization of CHO Cells by the Classical and Alternative Pathways

**Figure 2:**
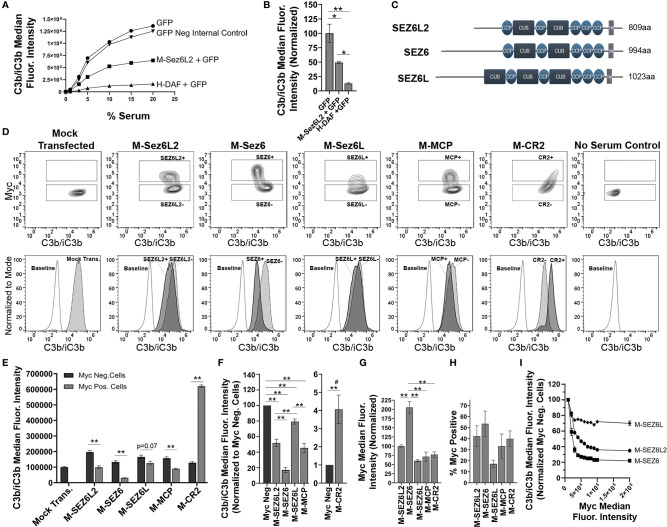
Full Length Sez6L2, Sez6, and Sez6L inhibit C3b/iC3b opsonization of CHO cells by the classical pathway. **(A, B)** Sez6L2 inhibits C3b/iC3b opsonization at a range of serum concentrations. CHO cells were transfected with plasmids for GFP alone or with Myc-tagged Sez6L2 (M-Sez6L2) or His-tagged DAF (H-DAF). CHO cells were coated with antibodies and exposed to 0-20% C5-depleted human serum for one hour and then immuno-stained with anti-C3b/iC3b antibodies and analyzed by flow cytometry. One experiment is shown that is representative of two independent experiments. **(B)** C3b/iC3b on GFP transfected cells with or without M-Sez6L2 or H-DAF at 15% serum. ANOVA (P=0.0016; F(2,6)=22.51). N=3; one experiment with three replicates (representative of 3+ independent experiments). **(C)** Schematic of Sez6L2, Sez6, and Sez6L protein domain structures. **(D–I)** CHO cells were transfected with the indicated Myc-tagged cDNAs and processed as outlined in A with 15% C5 depleted serum, except that an anti-Myc antibody was used in place of GFP to identify transfected and expressing CHO cells. **(D)** 5% Contour plots of C3b/iC3b versus Myc fluorescence (top layer) and C3b/iC3b fluorescence histograms (bottom layer) of the same samples normalized to mode and compared to baseline cells not exposed to serum. For Contour plots, boxed regions highlight cells designated as Myc-positive (top box) and Myc-negative (lower box) populations. For C3b/iC3b histograms, dark grey, solid line population = Myc-positive cells; Light grey, dotted line population= Myc-negative cells; White, dashed grey line population = baseline. Representative of 4+ independent experiments. **(E)** Quantification of the average median C3b/iC3b fluorescence intensity from Myc-positive and Myc-negative cells within each sample. Statistics = t-tests. N=3 (one experiment with three replicates; Representative of 4+ independent experiments). **(F)** Average median C3b/iC3b fluorescence intensities after normalization to the Myc-negative cells from each experimental group. ANOVA between Myc-positive cell populations (p<0.001; F(4, 15)=64.53). Sez6L2 inhibits C3b/iC3b opsonization at a level comparable to positive control MCP. Sez6 is a stronger complement inhibitor than Sez6L2 and Sez6L is a weaker inhibitor. **(F)** Average median Myc fluorescence intensity from Myc-positive cells. ANOVA (p<0.001; F(4, 15)=36.79). **(G)** Average % of Myc-positive cells in each experimental group (ANOVA, p=0.115; F(4, 15)=2.224). For sections **(F–H)**, N=4 (four independent experiments). **(I)** Sez6 blocks complement opsonization more efficiently than Sez6L2 and Sez6L even when comparing similar levels of Myc surface expression. Average C3b/iC3b median fluorescence intensity normalized to internal Myc-negative populations for M-Sez6, M-Sez6L2, and M-Sez6L samples shown relative to the Myc median fluorescence intensity. N=3 (one experiment with three replicates, Representative of three independent experiments). For all graphs *p < 0.05; **p < 0.01; ^#^p < 0.001 for all Myc-positive groups compared to M-CR2.

After having identified the Sez6 family as potential complement regulators due to their domain structure, we sought experimental evidence to support this putative function. First, we examined whether Sez6L2 expressed on CHO cells could reduce the amount of C3b/iC3b on the cell surface generated by the classical pathway. For the first set of experiments we used cells expressing the complement inhibitor, DAF, as a positive control. CHO cells were transfected with GFP alone or co-transfected with GFP and Myc-tagged Sez6L2 (M-Sez6L2) or His-tagged DAF (H-DAF). The cells were coated in antibodies and then exposed to C5-depleted human serum ranging in concentration from 0-20%. C5-depleted serum was used to prevent cell lysis, but still allow full C3b deposition by the classical pathway, which was detected with antibodies to C3b/iC3b. GFP positive cells co-transfected with M-Sez6L2 showed ~50% reduction of C3b/iC3b at all serum levels compared to CHO cells transfected with GFP alone (at 15% serum p=0.007, Holm-Sidak MCT) (**Figures 2A, B**). Cells expressing H-DAF reduced C3b/iC3b opsonization to ~13-15% compared to GFP alone (at 15% serum p=0.001). Thus, Sez6L2 does have complement inhibitory activity.

Next we compared the complement inhibitory activity of Sez6L2 to its family members Sez6 and Sez6L to learn whether complement inhibition is a family function or limited to Sez6L2. Sez6 and Sez6L are 49% and 50% identical to Sez6L2 respectively (**Figure 2C**). We also compared the Sez6 family members to two other Myc-tagged CCP domain-containing proteins, MCP and CR2. MCP is a transmembrane complement inhibitor composed of four CCP domains (61, 62). CR2 is a transmembrane protein composed of 15-16 tandem CCP domains, but it is not a complement inhibitor. However, CR2 does binds C3d/C3dg to present complement opsonized antigens to the adaptive immune system as well as initiate signal transduction cascades (63). It can also enhance C3b deposition by the alternative pathway on expressing cells (64, 65) and serves as a control in this paradigm for what excessive complement opsonization looks like. Mock transfected cells and cells without serum exposure were used as controls for full complement activation or a negative baseline respectively. First we assayed the amount of C3b/iC3b on cells initiated primarily by the classical pathway. M-Sez6L2, M-Sez6, M-Sez6L, and M-MCP all significantly decreased C3b/iC3b and M-CR2 significantly increased C3b/iC3b on expressing cells compared to non-transfected, Myc-negative cells within each experimental sample (**Figures 2D–F**). Overall, M-Sez6 was the most effective at decreasing C3b/iC3b opsonization as Myc-positive cells had only 17 ± 3% of the C3b/iC3b found on the Myc-negative cells in the same sample (p<0.001, Holm-Sidak MCT). M-Sez6L2 expressing cells had 52 ± 5% of C3b/iC3b compared to Myc-negative cells (p<0.001), M-Sez6L had 79 ± 3% (p=0.004), and M-MCP had 45 ± 6% (p<0.001). On the other hand, M-CR2 had 407 ± 78% the level of C3b/iC3b of internal Myc-negative cells P<0.001) (**Figure 2F**). These results show that Sez6 family members share the complement inhibitory function but have different levels of activity. Sez6 is the most effective inhibitor of the classical pathway. Sez6L2 is a moderate inhibitor and functions at a level comparable to MCP. Sez6L is a weak inhibitor.

Because M-Sez6 expressed on the cell surface at twice the level of Sez6L2, Sez6L, and MCP (**Figure 2G**), but usually in a similar percentage of cells (**Figure 2H**), we wondered whether M-Sez6 was a more effective inhibitor simply because of the higher expression levels or whether there are intrinsic activity differences in the family members. Thus we gated the cell populations based on increasing levels of Myc surface expression to compare C3b/iC3b opsonization levels in cells with similar levels of surface M-Sez6, M-Sez6L or M-Sez6L2. The results suggest that all Sez6 family members inhibit complement better with higher surface expression. However, M-Sez6 was still more effective at limiting C3b/iC3b opsonization than M-Sez6L and M-Sez6L2 at all expression levels (**Figure 2I**).

**Figure 3:**
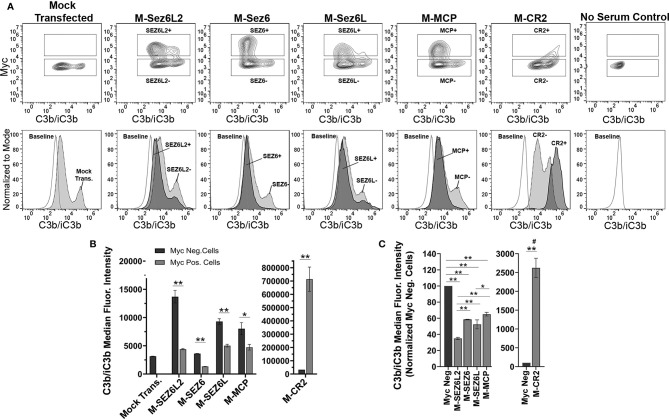
Full Length Sez6L2, Sez6, and Sez6L inhibit C3b/iC3b opsonization of CHO cells by the alternative pathway. CHO cells were transfected with the indicated Myc-tagged cDNAs and then coated with a low level of antibodies and exposed to 20% C5-depleted human serum for one hour in the presence of 10 mM EGTA and 10 mM MgCl_2_ to block the classical pathway. Cells were then labeled with anti-C3b/iC3b and anti-Myc antibodies and analyzed by flow cytometry. **(A)** 5% Contour plots of C3b/iC3b versus Myc fluorescence (top layer) and C3b/iC3b fluorescence histograms (bottom layer) of the same samples normalized to mode and compared to baseline cells not exposed to serum. For Contour plots, boxed regions highlight cells designated as Myc-positive (top box) and Myc-negative (lower box) populations. For C3b/iC3b histograms, dark grey, solid line population = Myc-positive cells; Light grey, dotted line population= Myc-negative cells; White, dashed grey line population = baseline. Representative of 3+ independent experiments with technical replicates. **(B)** Quantification of the average median C3b/iC3b fluorescence intensity from Myc-positive and Myc-negative cells within each sample. N=3 (one experiment with three replicates; Representative of 3+ independent experiments) Statistics = t-tests. E) Average median C3b/iC3b fluorescence intensities after normalization to the Myc-negative cells from each experimental group. ANOVA (p<0.001; F(4, 10)=74.47. N=3 (3 independent experiments). For all graphs *p < 0.05; **p < 0.01 ^#^p < 0.001 for all Myc-positive groups compared to M-CR2.

Next, full-length Sez6L2, Sez6, and Sez6L were analyzed for their ability to inhibit complement opsonization initiated primarily by the alternative pathway in the presence of Mg-EGTA. The results were similar to that obtained with the classical pathway and showed all Sez6 family members to be effective inhibitors of alternative pathway C3b/iC3b opsonization. However, their activities relative to each other were different (**Figures 3A–C**). M-Sez6L2 expressing cells had 34 ± 1%, M-Sez6 had 59 ± 2%, M-Sez6L had 52 ± 6%, M-MCP had 65 ± 2% and M-CR2 had 2624 ± 256% the level of deposited C3b/iC3b compared to Myc-negative cells within the same samples (**Figure 3C**). C3b/iC3b opsonization by the alternative pathway was less intense than the classical pathway and resulted in C3b/iC3b coated cells segregating into two main population peaks. The primary peak population had low C3b/iC3b opsonization. The second peak population contained less cells than the primary peak, but these cells were opsonized with high levels of C3b/iC3b that were almost equivalent to the levels found in the classical pathway assays. M-Sez6L2, M-Sez6, Sez6L, and M-MCP all prevented expressing cells from reaching the complement opsonization levels of the high C3b/iC3b peak population (**Figure 3A**). Alternatively, M-CR2 increased C3b/iC3b levels on expressing cells, shifting all Myc-positive cells into the high C3b/iC3b opsonized population. Interestingly, M-Sez6L2 expressing cells also had decreased C3b/iC3b levels in the lower peak population relative to the non-transfected, Myc-negative cells within the same sample (**Figures 3A, B**). However, these Myc-negative cells in M-Sez6L2 samples often had increased C3b/iC3b opsonization compared with the Myc-negative cells in other samples raising the question of whether M-Sez6L2 protects expressing cells at the expense of promoting complement opsonization on non-expressing cells. Nevertheless, the cells expressing Sez6 family members had less complement opsonization by the alternative pathway than non-expressing cells and their complement inhibitory activity equaled or exceeded the activity of the known complement regulator MCP. In summary, Sez6 family members are inhibitors of C3b/iC3b complement opsonization by both the classical and alternative pathways, but individual Sez6 family members vary in the efficacy of their complement inhibitory activity toward each pathway.

### Recombinant Sez6L2-MH Is a Soluble, Truncated Form of Sez6L2 With a Myc-6xHis Tag

**Figure 4:**
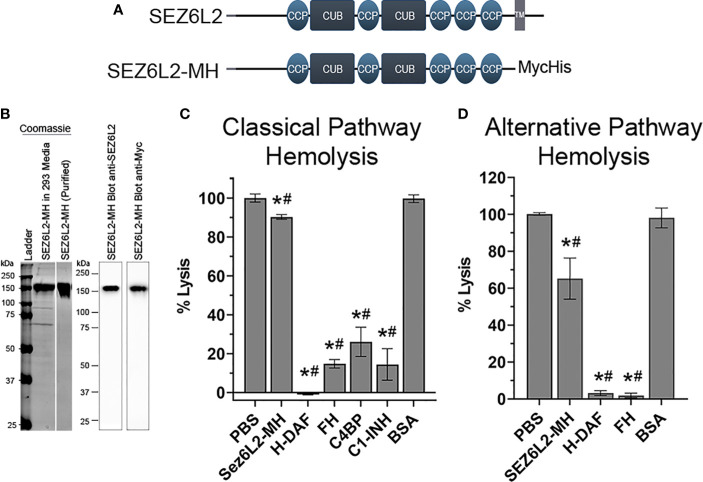
Truncated Sez6L2 inhibits alternative pathway hemolysis more than classical pathway hemolysis. **(A)** Schematic of Sez6L2 and Sez6L2-MH domain structures. CCP=Domain abundant in complement control proteins. CCP domains are also known as SUSHI repeats or short complement-like repeat (SCR). CUB= Domains named after complement C1r/C1s, uEGF, and BMP1. TM=Transmembrane region. Sez6L2-MH was made by replacing the transmembrane and cytoplasmic tail domains with a tandem Myc, 6xHis tag. **(B)** Purified Sez6L2-MH is shown by a Coomassie stained gel and by western blot with anti-Sez6L2 and anti-Myc antibodies. Lanes with the Coomassie stain are from the same gel. **(C)** Classical pathway hemolysis assay. Antibody-coated sheep erythrocytes were exposed to human serum pre-incubated with purified Sez6L2-MH, H-DAF, FH, C4BP, C1-INH, or BSA. After 30 mins, the percent of cell lysis was measured by spectrophotometry (A415). One-way ANOVA (P <0.0001; F(6,30)=233.2); PBS N=10; Sez6L2-MH N=7; H-DAF N=4; CFH N=4; C4BP N=3; C1-INH N=3; BSA N=6. **(D)** Alternative pathway hemolysis assay. Rabbit erythrocytes were exposed to human serum pre-incubated with Sez6L2-MH, complement regulators, or BSA in presence of 10 mM MgEGTA to block the classical pathway. Then the percent of cell lysis was measured by spectrophotometry (A415). One-way ANOVA (P <0.0001; F(4,16)=33.88). PBS N=6, Sez6L2-MH N=5; FH N=2, H-DAF N=2, and BSA N=6. For all graphs *p < 0.05 compared to PBS control and ^#^p < 0.05 compared to BSA negative control.

In order to determine the specific mechanisms by which Sez6L2 inhibits complement using traditional assays, we generated a truncated expression construct of human Sez6L2, named Sez6L2-MH, which replaced the transmembrane domain and cytoplasmic tail with a tandem Myc-6xHis tag. This plasmid was expressed in HEK293 cells grown in serum free media and the secreted protein was purified *via* the His-tag. The purity and identity of the Sez6L2-MH protein were verified by SDS-PAGE under reducing conditions followed by Coomassie staining and anti-Sez6L2 or anti-Myc western blots (**Figures 4A, B**).

### Sez6L2-MH Partially Inhibits Alternative Pathway Hemolysis and Only Modestly Inhibits the Classical Pathway Hemolysis

Complement activation by the classical and alternative pathways ultimately results in the formation of the membrane attack complex which directly lyses target cells. As such, complement-mediated lysis of erythrocytes, also known as hemolysis, is a useful assay to measure the ability of Sez6L2-MH to regulate the complement pathway. We tested Sez6L2-MH alongside complement inhibitory proteins FH, C4BP, and C1-INH purified from human serum and His-tagged DAF (H-DAF) purified similarly to Sez6L2-MH. BSA was also tested as a negative control protein. Sez6L2-MH was able to only modestly inhibit complement-mediated lysis of antibody-coated sheep erythrocytes by the classical pathway, exhibiting 90.4 ± 1.2% lysis compared to 100.0 ± 2.1% lysis by buffer alone (p=0.013; Holm-Sidak MCT; **Figure 4C**). The other complement inhibitory proteins efficiently and significantly prevented lysis (H-DAF: -1.0 ± 0.2%; FH: 14.8 ± 2.2%; C4BP 26.1 ± 7.5%; C1-INH: 14.4 ± 8.1% lysis). As expected, BSA had no effect on erythrocyte lysis by the classical pathway (100.2 ± 3% lysis).

A similar hemolysis assay was used for the alternative pathway and was performed in the presence of EGTA. The initiating steps of the classical and lectin pathways requires calcium, which is preferentially chelated by EGTA, effectively preventing classical and lectin pathway initiation while sparing the alternative pathway. Sez6L2-MH was able to partially inhibit rabbit erythrocyte lysis by the alternative pathway, showing 65.2 ± 11.1% lysis compared to 100 ± 0.7% lysis by buffer alone (p<0.001; **Figure 4D**). Comparatively, H-DAF and FH fully prevent lysis to 3.2 ± 1.3% and 1.9 ± 1.2% respectively. In contrast, the negative control BSA resulted in no change in erythrocyte lysis compared to buffer alone (98.1 ± 5.3%). In summary, recombinant Sez6L2-MH is a weak inhibitor of hemolysis mediated by the classical pathway and a moderate inhibitor of hemolysis mediated by the alternative pathway.

### Sez6L2-MH Functions as a Cofactor for Fluid-Phase Factor I (FI) Cleavage of C3b but Not C4b

**Figure 5:**
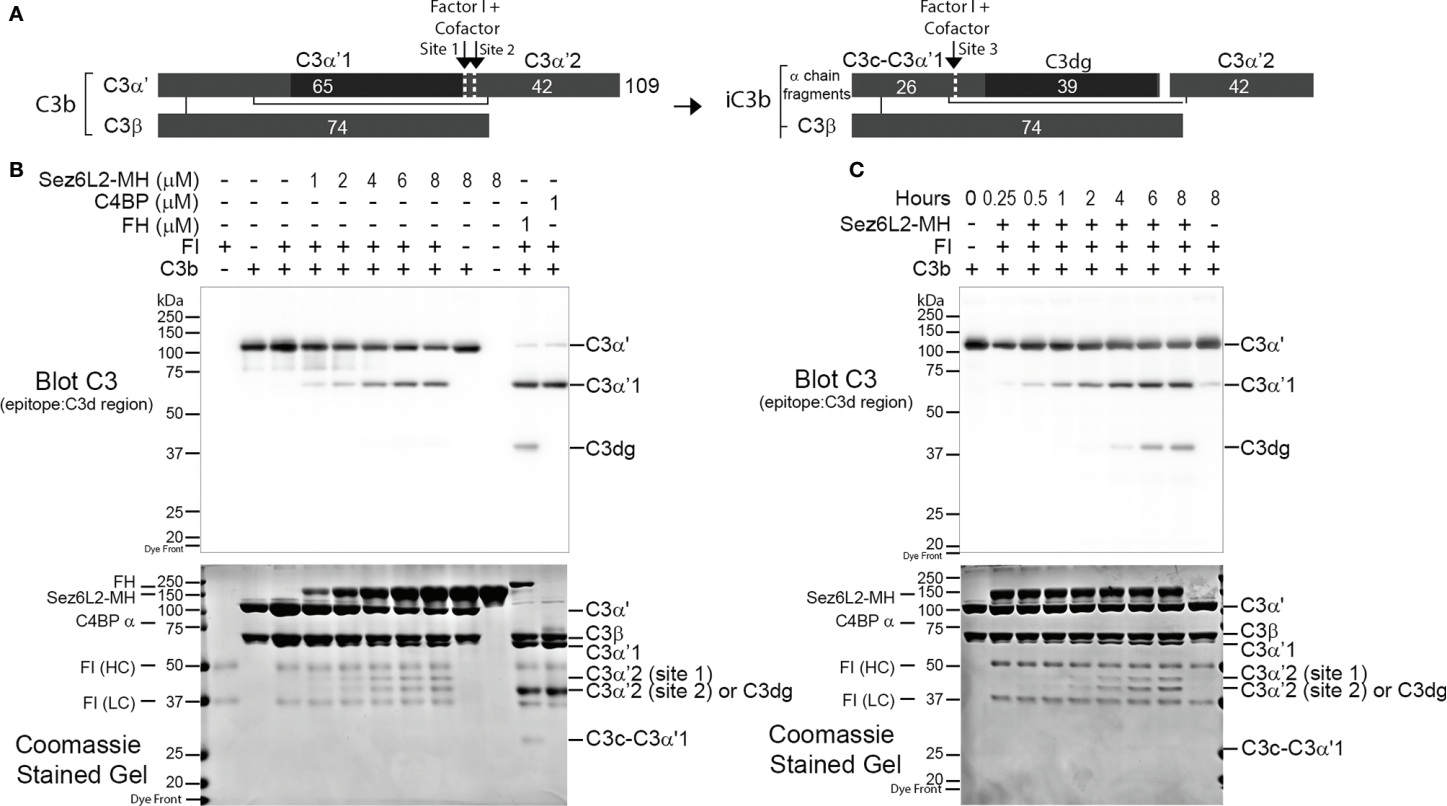
Truncated Sez6L2 is a cofactor for Factor I cleavage of C3b. **(A**) Schematic of Factor I and cofactor cleavage of C3b and iC3b. **(B)** C3b and Factor I (FI) were incubated alone, with concentrations of Sez6L2-MH ranging from 1 to 8 µM, or with 1 µM Factor H (FH) or C4BP for two hours at 37°C. Then samples were analyzed by western blot using antibodies that recognize C3d, a region within the C3α chain [and highlighted by the black rectangle in the schematics in **(A)**]. Coomassie stained gels are also shown. Sez6L2-MH supports partial FI cleavage of C3b at sites 1 and 2 generating the cleavage products C3α’1 and C3α’2 in a concentration dependent manner. FH and C4BP (known co-factors of FI towards C3b) supported almost full cleavage of C3b at sites 1 and 2. FH also showed partial cleavage at site 3 generating some C3dg and C3c-C3α’1. **(C)** C3b and FI were incubated with 1.5 µM Sez6L2-MH for 0.25-8 hours and then were visualized by western blot and Coomassie stained gels. Sez6L2-MH facilitates more FI cleavage with increased time including some partial cleavage at site 3 to generating C3dg and C3c-C3α’1.

We next investigated whether recombinant Sez6L2-MH functions as a cofactor for Factor I cleavage of C3b. FH and C4BP are known co-factors of FI towards C3b (66–70) and were used as positive controls. C3b is composed of the C3α’ chain and the C3β chain linked by disulfide bonds (see schematic in **Figure 5A**). With cofactor support, FI cuts C3b sequentially at site 1 (Arg^1303^-Ser^1304^), then site 2 (Arg^1320^-Ser^1321^) and finally site 3 (Arg^954^-Glu^955^; UniProt numbering of prepro-C3) (68). Cofactors can support efficient cleavage at one, two, or all three sites (68–70). For example, FH facilitates efficient cleavage at sites 1 and 2, but has very weak and slow cofactor function for site 3 [(68–70); and **Figure 5B**]. Incubation of C3b (1.5 µM) and FI (0.5 µM) with 1 µM FH or C4BP for 2 hours resulted in an almost complete loss of C3α’ and the appearance of two cleavage products: C3α’1 (visible by western blot with a C3d antibody and in the Coomassie stained gel) and C3α’2-(cut at site 2; visible in the Coomassie stained gel as a ~42 kDa band) (**Figure 5B**). A small amount of C3dg and C3c-C3a’1 also appeared in the FH sample, signaling some cleavage at site 3. FI incubated with C3b and increasing amounts of Sez6L2-MH (1 µM-8 µM) for two hours facilitated partial FI cleavage of the C3α’ chain to C3α’1 and C3α’2 in a dose-dependent manner. Sez6L2-MH facilitated cleavage of C3b at site 1 and less efficient cleavage of C3b at the second cleavage site generating both the larger C3α’2-site 1 band (~44 kDa) and the smaller C3α’2-site 2 band (~42 kDa). We also assayed Sez6L2-MH cofactor activity occurring at incubation times ranging from 15 minutes to 8 hours. 1.5 µM of Sez6L2 facilitated some FI cleavage of C3b at site 1 starting at 15 minutes. Longer incubation times yielded increasing amounts of C3b cut by FI at sites 1 and 2 (**Figure 5C**). With incubation times at four hours or longer, Sez6L2-MH also facilitated a small amount of cleavage at site 3, generating some C3dg and C3c-C3α’1. Incubation of C3b with Sez6L-MH alone did not result in any C3b cleavage products, indicating that FI is the active protease responsible for C3b cleavage in the presence of Sez6L2-MH (**Figure 5B**).

**Figure 6:**
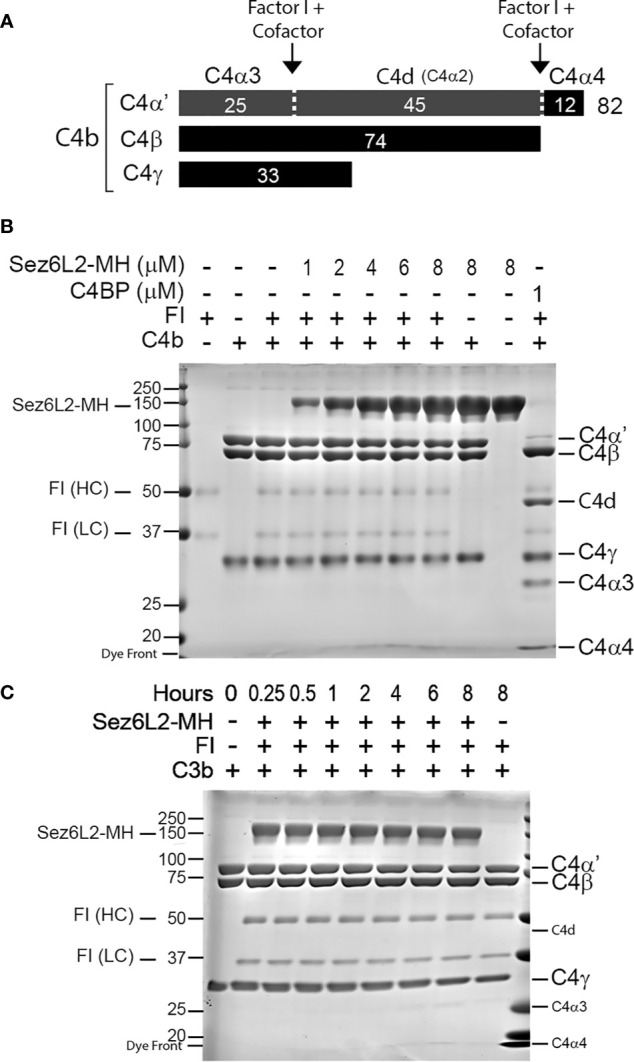
Truncated Sez6L2 does not act as a cofactor for Factor I cleavage of C4b. **(A)** Schematic of Factor I plus cofactor cleavage of C4b. **(B)** C4b and FI were incubated alone, with concentrations of Sez6L2-MH ranging from 1 to 8 µM, or with 1 µM C4BP for two hours at 37°C. Then samples were then run and visualized on Coomassie stained gels. Incubation of C4b and FI with Sez6L2-MH did not result in the appearance of C4b cleavage products C4d, C4α3, or C4α4. On the other hand, C4BP (a known cofactor of FI for C4b cleavage) supported FI’s production of C4d, C4α3, and C4α4. **(C)** Sez6L2-MH does not support Factor I cleavage of C4b even with increased time. C4b and FI were incubated with 1.5 µM Sez6L2-MH for 0.25-8 hours and then visualized on Coomassie stained gels.

Some FI cofactors are specific for either C3b or C4b, while others work on both. Therefore, the FI cleavage assay was repeated with C4b. C4b is composed of three peptide chains—C4α’, C4β, and C4γ—attached by disulfide bonds. Cofactors aid FI to cut the C4α’ chain at two locations yielding C4d, C4α3, and C4α4 (see schematic in **Figure 6A**). C4b (1.5 µM) and FI (0.5 µM) were incubated alone, with 1 µM of C4BP, or with concentrations of Sez6L2-MH ranging from 1 to 8 µM for two hours. C4BP was able to facilitate almost complete cleavage of C4α’ to C4α3, C4α4, and C4d. No amount of Sez6L2-MH tested was able to promote any FI cleavage of C4b (**Figure 6B**). Longer incubation times up to eight hours also did not allow Sez6L2-MH to promote FI cleavage of C4b (**Figure 6C**). In summary, Sez6L2 is a cofactor for FI that facilitates partial cleavage of C3b, but not C4b.

### Sez6L2-MH Accelerates the Decay of C3 Convertases

**Figure 7:**
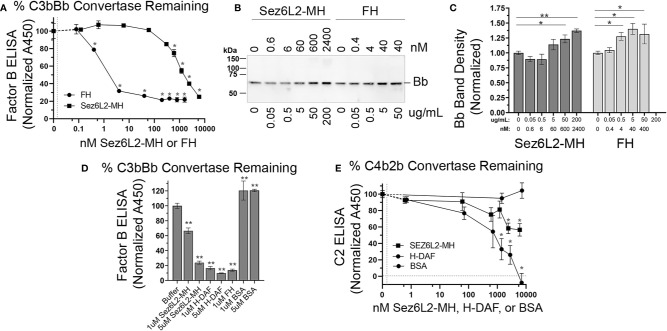
Truncated Sez6L2 has decay accelerating activity for the alternative pathway C3 convertase but has only modest decay accelerating activity for the classical/lectin pathway C3 convertase. **(A–D)** Alternative C3 convertase assay: **(A–C)** A 96 well plate coated with C3b was incubated with Factor B and Factor D to form the C3 convertase C3bBb, then incubated with Sez6L2-MH or FH at various concentrations ranging from 0-6000 nM (equivalent to 0-500 µg/mL) to assess their decay accelerating activity. Factor B remaining bound to the plate (as C3bBb) was detected using an anti-Factor B antibody ELISA in **(A)** and Bb released into the supernatant is shown *via* western blot in **(B)** with Bb band densities quantified in **(C)**. For the western blot and quantification, Sez6L2-MH and FH concentrations are listed in both nM and µg/mL. Quantification of Bb band densities were normalized to the 0 nM control lanes. N=3 samples per group. **(D)** The alternative C3 convertase decay ELISA described above was repeated comparing 0, 1uM, and 5uM of Sez6L2-MH, H-DAF, FH, and BSA. **(E)** Classical C3 convertase assay: A plate coated with C4b was incubated with C2 and C1s-enzyme to form the classical/lectin pathway C3 convertase C4b2b, then incubated with Sez6L2-MH, H-DAF at concentrations ranging from 0 to 7000 nM to assess their decay accelerating activity. C2 remaining bound to the plate (presumably as C4b2b) was detected using an anti-C2 antibody ELISA. For A, D, and E ELISAs: N=3 (1 experiment with 3 replicates; representative of 2-3 independent experiments). Statistics: one-way ANOVAS with Holms-Sidak multiple comparison’s tests to controls. *p < 0.05, **< 0.01.

Complement inhibitors that act at the level of C3 convertases, function not only as cofactors for FI, but they can also act as decay accelerating factors to irreversibly dissociate the catalytic subunit (Bb/C2b) from the non-catalytic subunit (C3b/C4b). MCP functions primarily as a cofactor and does not have decay accelerating activity. Alternatively, DAF was named for its decay accelerating activity and has no cofactor function. FH has both cofactor and decay accelerating activity (36). To investigate whether Sez6L2-MH has decay accelerating activity for the alternative pathway C3 convertase, we coated C3b on a 96 well plate and then added Factor B and Factor D to generate C3bBb bound to the plate. Sez6L2-MH or FH were added to the wells in increasing concentrations. The ability of Sez6L2-MH or FH to displace Bb from the bound C3bBb complex was assessed in an ELISA assay using an anti-FB antibody. The percent of FB remaining on the plate (normalized to buffer only control) was graphed against the concentrations of FH and Sez6L2-MH. Sez6L2-MH at lower concentrations (0.1 nM-60 nM) had no effect on the dissociation of Bb from the C3bBb convertase complex, while higher concentrations of Sez6L2 (600 nM-6 µM) significantly decreased the amount of FB on the plate by 25-72% in a dose dependent manner (**Figure 7A**). FH was effective at much lower concentrations, as 0.4 - 40 nM significantly decreased the amount of FB on the plate by 22-74%. Higher concentrations of FH (200 nM – 1.6µM) were also effective and reduced the convertase associated FB by just over 75% (a point at which the maximal decay activity appeared to plateau). The relative IC50 for FH was between 1-2 nM and the relative IC50 for Sez6L2-MH was ~1.2 µM.

Supernatants collected from the plate at the end of the incubation with Sez6L2-MH or FH were collected and analyzed by western blot with antibodies to Factor B. With increasing levels of Sez6L2-MH or FH, we found increasing levels of Bb (MW 70 KDa) in the supernatant, which mirror the deceasing amounts of Factor B bound to the ELISA plates (**Figures 7B, C**). We did not detect any full-length FB in the supernatants which shows that the decreasing plate-associated FB detected in the ELISA was mostly in the Bb form. We additionally compared the C3bBb decay activity of Sez6L2-MH and FH to that of H-DAF and a negative control protein, BSA at 0, 1, and 5 µM (**Figure 7D**). At 1µM, the decay activity of H-DAF and FH reduced the plate associated FB by 83% and 87% respectively, while 5 µM DAF reduced FB by 90.1%. Whereas, at 1 µM, Sez6L2-MH reduced plate associated FB by 44% and at 5 µM Sez6L2-MH reduced FB to 76% compared to the buffer only control and the BSA negative controls at 1 µm and 5 µM (p<0.001 for all). Taken together, these data show that soluble Sez6L2-MH exhibits decay accelerating activity for the alternative pathway C3 convertase C3bBb, but requires higher concentrations than FH and H-DAF.

Next we tested whether Sez6L2-MH could accelerate the decay of the classical/lectin pathway C3 convertase, C4b2b. A plate coated with C4b was incubated with C2 and C1s-enzyme to form the C3 convertase, C4b2b, then incubated with Sez6L2-MH or H-DAF at concentrations ranging from 0 to 7 µM to assess their decay accelerating activity. C2 remaining bound to the plate (presumably as C4b2b) was detected using an anti-C2 antibody ELISA (**Figure 7E**). H-DAF effectively removed bound C2b from the plate in a dose dependent manner with 7 µM H-DAF resulting in complete dissociation of C2b. Sez6L2-MH was also able to dissociate the C2b in a dose dependent manner with the maximal dose of 6 µM Sez6L2-MH reducing C2b by 43% (plate bound C2b with Sez6L2-MH = 57 ± 8% % vs 100 ± 4% % with buffer alone; p=0.002 Holm-Sidak MCT). Thus Sez6L2-MH has only modest decay accelerating activity for the classical/lectin pathway C3 convertase.

## Discussion

We have shown that Sez6, Sez6L, and Sez6L2 are all novel complement regulators that inhibit C3b/iC3b opsonization by the classical and alternative pathways. Sez6 was the most effective inhibitor of the classical pathway and functioned at a level equivalent to H-DAF and better than MCP. It also provided strong protection against the alternative pathway. Sez6L2 was a moderate inhibitor for the classical pathway that performed at a level similar to MCP and Sez6L2 was perhaps the most protective family member against the alternative pathway. Sez6L was a weak inhibitor towards the classical pathway but also functioned similar to MCP against the alternative pathway. We additionally showed that a truncated and soluble version of Sez6L2 partially prevented hemolysis by the alternative pathway, but only modestly blocks hemolysis by the classical pathway. Sez6L2 does this by inhibiting C3 convertases. Specifically, Sez6L2 inhibits complement by accelerating the decay of the alternative pathway C3 convertase, C3bBb, but has only weak decay activity toward the classical pathway convertase, C4b2b. Sez6L2 also functions as a cofactor for Factor I to facilitate cleavage of C3b, but not C4b. While most of our data do not employ neuronal specific paradigms, we have used well established methods of complement activity in serum to ascertain novel Sez6 family functions that are still relevant to complement in the brain or other tissues.

Previous studies have shown that Sez6 family proteins control dendritic branching and synaptic density by unknown mechanisms (13, 15, 16, 24). Additionally, genetic loss of the entire Sez6 family results in impaired motor coordination and motor learning, and impaired cognition (13–16). Sez6L2 proteins can be found throughout the somatodendritic compartment in some neuronal cell types like Purkinje cells of the cerebellum (14, 15). However, we report here that Sez6L2 can also be localized to synapses as we found in the CA1 region of the hippocampus. This synaptic location is also in line with a proteomic screen on dissociated cortical neurons reported by Loh et al. that found Sez6L2 within the excitatory synaptic cleft (71). The synaptic and dendritic localization of Sez6L2 puts it in an ideal location to protect synapses and dendrites from complement-dependent pruning during development and may be a mechanism by which Sez6 proteins modulate synapse numbers and dendritic morphology. However, Sez6 proteins could also modulate complement activation levels affecting neurogenesis, neuronal migration, or immune reactions to various insults. Perhaps complement dysregulation explains the genetic association of the Sez6 family with multiple neurodevelopmental and psychiatric disorders including: autism, schizophrenia, intellectual disability, epilepsy, and bipolar disorder (1–9).

We found that both full-length and truncated versions of Sez6L2 were capable of inhibiting complement. This is likely important as the extracellular domains of all Sez6 family proteins can be cut near the transmembrane region by BACE enzymes (1 and 2) (16, 72, 73). This provides a means to remove the complement inhibitors from the cell surface making the cell vulnerable to complement opsonization and its downstream consequences. On the other hand, it also provides a mechanism to release soluble complement inhibitors that could be useful to nearby “self” surfaces or the CSF. Soluble Sez6 is elevated in the CSF of patients with schizophrenia, bipolar, major depression and inflammatory pain compared to controls (74, 75). However, soluble Sez6 is decreased in the CSF in Alzheimer’s (76). Thus, altered shedding of the extracellular complement regulatory region of Sez6 proteins is likely connected and may have direct consequences on the development of neuropathology. Cells may also change their vulnerability to complement by expressing alternative short splice variants of Sez6 family members that do not contain a transmembrane anchor and the three tandem CCP domains that presumably mediate complement inhibition (13, 15).

The truncated Sez6L2-MH purified protein was very useful in examining the specific mechanisms by which Sez6L2 inhibits complement at the level of the C3 convertases. However, we also found it was less effective than the positive controls used in our studies (FH, DAF, and C4BP). It is possible that the purification process led to partial deactivation of Sez6L2-MH, necessitating higher levels of Sez6L2-MH to achieve complement inhibition. It is also possible that the truncated version could be less active than the full-length transmembrane protein due to its inability to localize to areas of complement activation or to correctly orient the complement inhibitory domains for efficient inhibition. Naturally soluble serum regulators like FH and C4BP have multiple C3 binding sites or can oligomerize to enhance homing to areas of active complement deposition. They also have binding sites for other complement factors or “self” membranes that aid recruitment and correct orientation of the inhibitors. These properties may also help explain their superior activity compared to our recombinant, Sez6L2-MH. Furthermore, truncation of other complement inhibitors, like FH, CR1, MCP, or SUSD4 can result in reduced activity compared of the full-length proteins (36, 77–79). For example, soluble MCP barely functions as a cofactor for the cleavage of erythrocyte-bound C3b, but it is an efficient cofactor in its full-length, cell-bound form (79).

The domain structure of the Sez6 family which contains five CCP domains (three of which are consecutive) initially prompted us to investigate the complement regulatory functions of Sez6 family members. Although proteins with multiple CCP domains are common to the complement pathway, there are many other proteins that contain CCP domains (even tandem CCP domains) that do not have complement inhibitory functions. Although the amino acid sequences of CCP domains vary considerably, complement regulatory proteins bind to C3b using a common binding platform on C3b with their 3-4 consecutive CCP domains in an extended orientation (38). Ojha et al. recently used AI-assisted computer learning along with significant functional annotation based on decades of experimental research on complement regulatory proteins to propose five short motifs that potentially confer complement inhibitory activity when conserved in a specific order across three tandem CCP domains (29). Using their web-based CoreDo program, we found Sez6L2 has the correct motif pattern for predicted complement regulatory activity (29). Thus, our results on Sez6L2 match their prediction. However, Sez6 and Sez6L do not have the correct five motif pattern; and yet, Sez6 was even more efficient at inhibiting C3b/iC3b opsonization by the classical pathway than Sez6L2. Interestingly, CSMD1, another brain expressed complement inhibitor associated with neurodevelopmental disorders, and perhaps a distant cousin of the Sez6 family, was also one of only two experimentally validated complement inhibitors that did not fit the five-motif pattern identified and reported by Ojha et al. (29, 80–83). As the core CCP motif pattern is somewhat different between Sez6L2 and Sez6, it is possible that there are unique features to their complement regulatory activity yet to be discovered.

Our study focused on C3 convertase inhibitory activities that have long been associated with CCP-domain containing complement regulators. We have assumed that much of the complement inhibitory activity of Sez6 family proteins comes from the sub-region with three adjacent CCP domains. However, CUB domains are also known to mediate multiple protein-protein interactions within the complement pathway, including the binding of C1r/C1s to C1q (84, 85). Therefore, the CUB domains of Sez6 proteins may mediate more interactions with complement factors in order to boost, broaden, or limit their impact on the complement pathways. Interestingly, different splice variants of the Sez6 family yield proteins with either two or three CUB domains. The Sez6L variant we tested contained three CUB domains and was the least effective family member at blocking C3 deposition by the classical pathway. In contrast, the variants of Sez6 and Sez6L2 we tested had only two CUB domains and were much more effective at blocking the classical pathway. Future studies will investigate whether the C3 convertase inhibitory activities can be isolated to the region of Sez6 proteins containing three adjacent CCP domains and will additionally seek to determine whether the CUB domains positively or negatively modulate the complement inhibitory activity of the Sez6 family members.

These studies were motivated by our overarching goal to better understand the identity and role of complement regulatory proteins in neurodevelopment and neurodegeneration. Yet, the complement inhibitory activity of the Sez6 family may also explain their role in cancer. Increased expression of Sez6 family members has been linked to increased tumor growth and a poor prognosis in various cancers (17–23). The role of complement in tumorigenesis is complex as it may exacerbate or inhibit tumor growth depending on the immune response and inflammatory environment. However, increased expression of complement regulatory proteins has been repeatedly shown to limit innate immune surveillance and cytotoxicity by the complement system and provide resistance to antibody based immunotherapies (86). Increased expression of complement regulators may also dampen the B and T cell immune response to tumor cells after chemotherapy due to complement/immune cell crosstalk mediated *via* CR2 (86, 87). Thus complement resistance not only influences the course of the disease but also limits therapeutic options. Testing for increased Sez6 family expression and employing strategies to block their complement inhibitory function alongside other therapeutic approaches may be necessary as it has been against tumors overexpressing other complement regulators like MCP, DAF, FH, or CD59.

## Data Availability Statement

The raw data supporting the conclusions of this article will be made available by the authors, without undue reservation.

## Ethics Statement

Animal care and use were carried out in compliance with the US National Research Council’s Guide for the Care and Use of Laboratory Animals and the US Public Health Service’s Policy on Humane Care and Use of Laboratory Animals. The animal study was reviewed and approved by University Committee on Animal Resources at the University of Rochester.

## Author Contributions

JH designed the study. JH and HL performed the IHC. WQ, PS, and JH did the CHO cell/C3 deposition flow cytometry assays. WQ, SL, and JH did the hemolysis assays. SL, SM, and JH did the factor I cleavage assays. SM and JH did the decay accelerating assays. JH wrote the manuscript. JG provided expertise on the Sez6 family. All authors contributed to the article and approved the submitted version.

## Funding

This work was supported by funding from the National Institutes of Health (R21NS111255), the Harry T. Mangurian Jr. Foundation, the National Health and Medical Research Council (NHMRC, GNT1099930 and GNT1140050), and the Judith Jane Mason and Harold Williams Memorial Foundation.

## Conflict of Interest

The authors declare that the research was conducted in the absence of any commercial or financial relationships that could be construed as a potential conflict of interest.
