# Correction: Sez6L2 inhibits complement by facilitating factor I cleavage of C3b and accelerating the decay of C3 convertases

**DOI:** 10.3389/fimmu.2026.1872549

**Published:** 2026-05-21

**Authors:** Wen Q. Qiu, Shaopeiwen Luo, Stefanie A. Ma, Priyanka Saminathan, Herman Li, Jenny M. Gunnersen, Harris A. Gelbard, Jennetta W. Hammond

**Affiliations:** 1 Center for Neurotherapeutics Discovery, Department of Neurology, University of Rochester Medical Center, Rochester, NY, United States; 2 Department of Anatomy and Neuroscience and The Florey Institute of Neuroscience and Mental Health, University of Melbourne, Melbourne, VIC, Australia

**Keywords:** Sez6, Sez6L, Sez6L2, complement, classical pathway, alternative pathway, Factor I cofactor, C3 convertase

The title of this article was erroneously given as: “The Sez6 family inhibits complement by facilitating factor I cleavage of C3b and accelerating the decay of C3 convertases.”

The correct title of the article is “Sez6L2 inhibits complement by facilitating factor I cleavage of C3b and accelerating the decay of C3 convertases.”

There was a mistake in **Figures 2**, **3** as published. The Sez6 data was incorrect due to the use of a mislabeled plasmid. This applies only to the Sez6 data in **Figures 2D-I**, **3A-C**. The corrected **Figures 2**, **3** appear below.

**Figure 2:**
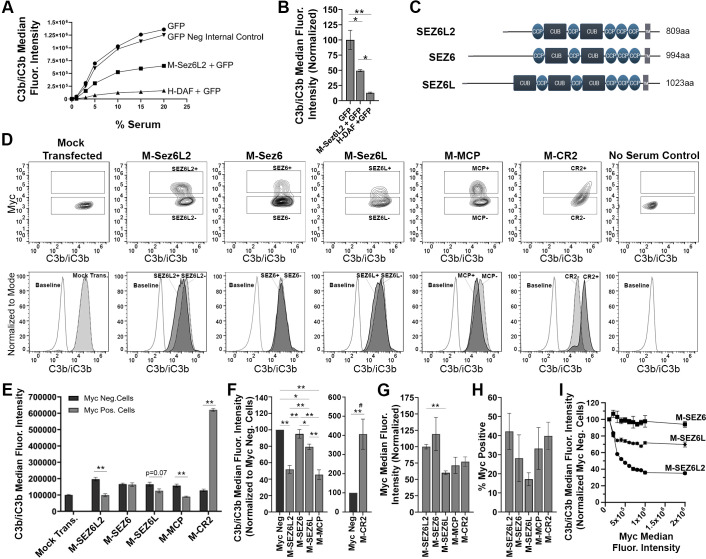
Full Length Sez6L2 and Sez6L inhibit C3b/iC3b opsonization of CHO cells by the classical pathway. **(A, B)** Sez6L2 inhibits C3b/iC3b opsonization at a range of serum concentrations. CHO cells were transfected with plasmids for GFP alone or with Myc-tagged Sez6L2 (M-Sez6L2) or His-tagged DAF (H-DAF). CHO cells were coated with antibodies and exposed to 0-20% C5-depleted human serum for one hour and then immuno-stained with anti-C3b/iC3b antibodies and analyzed by flow cytometry. One experiment is shown that is representative of two independent experiments. **(B)** C3b/iC3b on GFP transfected cells with or without M-Sez6L2 or H-DAF at 15% serum. ANOVA (P=0.0016; F(2,6)=22.51). N=3; one experiment with three replicates (representative of 3+ independent experiments). **(C)** Schematic of Sez6L2, Sez6, and Sez6L protein domain structures. **(D–I)** CHO cells were transfected with the indicated Myc-tagged cDNAs and processed as outlined in A with 15% C5 depleted serum, except that an anti-Myc antibody was used in place of GFP to identify transfected and expressing CHO cells. **(D)** Contour plots of C3b/iC3b versus Myc fluorescence (top layer) and C3b/iC3b fluorescence histograms (bottom layer) of the same samples normalized to mode and compared to baseline cells not exposed to serum. For Contour plots, boxed regions highlight cells designated as Myc-positive (top box) and Myc-negative (lower box) populations. For C3b/iC3b histograms, dark grey, solid line population = Myc-positive cells; Light grey, dotted line population= Myc-negative cells; White, dashed grey line population = baseline. Representative of 4+ independent experiments. **(E)** Quantification of the average median C3b/iC3b fluorescence intensity from Myc-positive and Myc-negative cells within each sample. Statistics = t-tests. N=3 (one experiment with three replicates; Representative of 4+ independent experiments). **(F)** Average median C3b/iC3b fluorescence intensities after normalization to the Myc-negative cells from each experimental group. ANOVA between Myc-positive cell populations (p<0.001; F(5, 18)=58.52). Sez6L2 inhibits C3b/iC3b opsonization at a level comparable to positive control MCP. Sez6 has no inhibitor activity and Sez6L is a weaker inhibitor. **(G)** Average median Myc fluorescence intensity from Myc-positive cells. Brown-Forsythe ANOVA (p<0.001; F*(5, 7.474)=15.26). **(H)** Average % of Myc-positive cells in each experimental group (ANOVA, p=0.354; F(4, 15)=1.192). For sections **(F–H)**, N=4 (four independent experiments). **(I)** Sez6L2 blocks complement opsonization more efficiently than Sez6L and Sez6 even when comparing similar levels of Myc surface expression. Average C3b/iC3b median fluorescence intensity normalized to internal Myc-negative populations for M-Sez6, M-Sez6L2, and M-Sez6L samples shown relative to the Myc negative median fluorescence intensity. N=3 (one experiment with three replicates, Representative of three independent experiments). For all graphs *p < 0.05; **p < 0.01; ^#^p < 0.001 for all Myc-positive groups compared to M-CR2.

**Figure 3:**
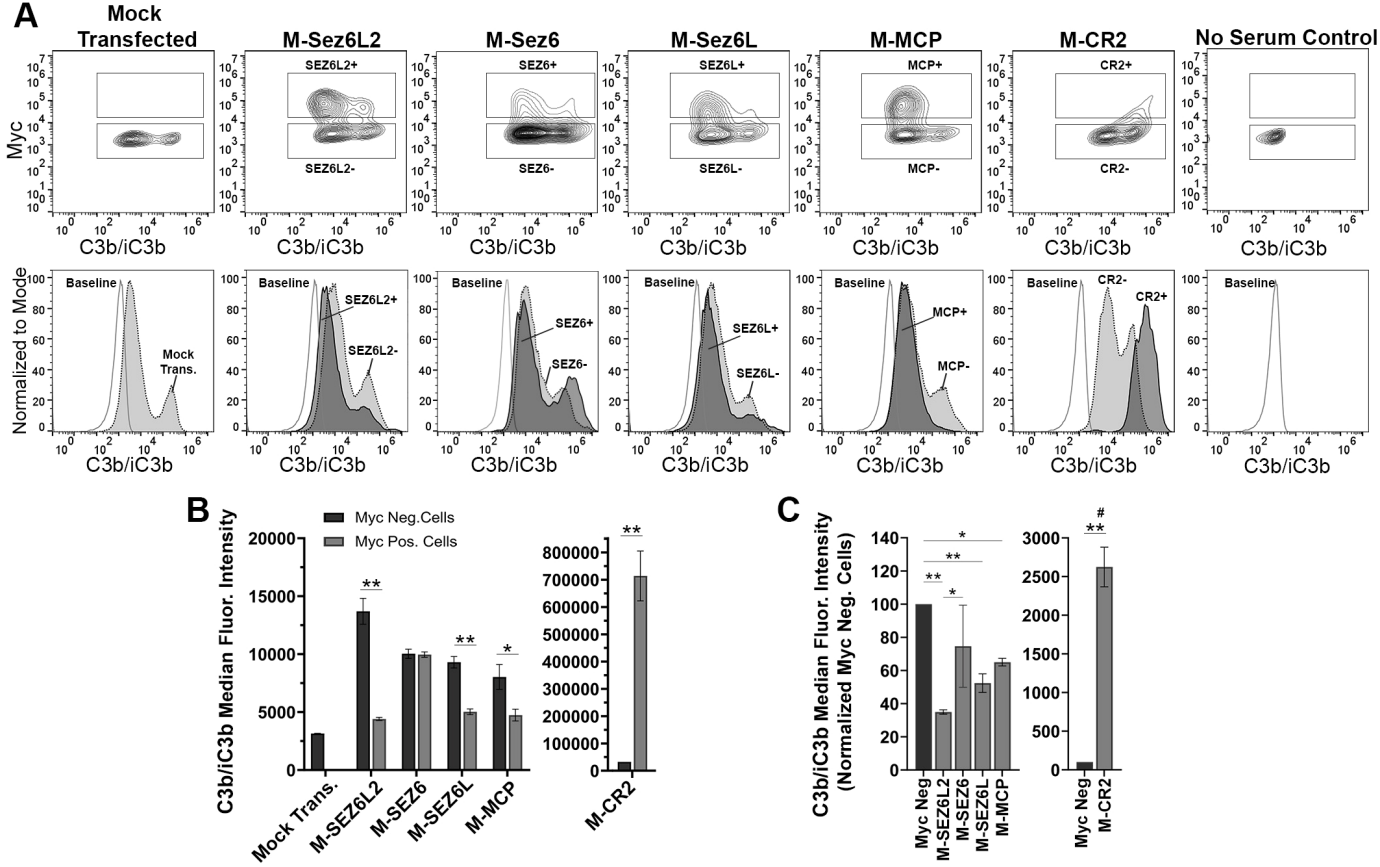
Full Length Sez6L2 and Sez6L inhibit C3b/iC3b opsonization of CHO cells by the alternative pathway. CHO cells were transfected with the indicated Myc-tagged cDNAs and then coated with a low level of antibodies and exposed to 20% C5-depleted human serum for one hour in the presence of 10 mM EGTA and 10 mM MgCl2 to block the classical pathway. Cells were then labeled with anti-C3b/iC3b and anti-Myc antibodies and analyzed by flow cytometry. **(A)** Contour plots of C3b/iC3b versus Myc fluorescence (top layer) and C3b/iC3b fluorescence histograms (bottom layer) of the same samples normalized to mode and compared to baseline cells not exposed to serum. For Contour plots, boxed regions highlight cells designated as Myc-positive (top box) and Myc-negative (lower box) populations. For C3b/iC3b histograms, dark grey, solid line population = Myc-positive cells; Light grey, dotted line population= Myc-negative cells; White, dashed grey line population = baseline. Representative of 3+ independent experiments with technical replicates. **(B)** Quantification of the average median C3b/iC3b fluorescence intensity from Myc-positive and Myc-negative cells within each sample. N=3 (one experiment with three replicates; Representative of 3+ independent experiments) Statistics = t-tests. **(C)** Average median C3b/iC3b fluorescence intensities after normalization to the Myc-negative cells from each experimental group. ANOVA (p<0.01; F(5, 11)=10.81. N=2-3 independent experiments. For all graphs *p < 0.05; **p < 0.01 ^#^p < 0.001 for all Myc-positive groups compared to M-CR2

There was a mistake in the caption of **Figure 2** as published. The original caption stated

“Full Length Sez6L2, Sez6, and Sez6L inhibit C3b/iC3b opsonization of CHO cells by the classical pathway. **(A, B)** Sez6L2 inhibits C3b/iC3b opsonization at a range of serum concentrations. CHO cells were transfected with plasmids for GFP alone or with Myc-tagged Sez6L2 (M-Sez6L2) or His-tagged DAF (H-DAF). CHO cells were coated with antibodies and exposed to 0-20% C5-depleted human serum for one hour and then immuno-stained with anti-C3b/iC3b antibodies and analyzed by flow cytometry. One experiment is shown that is representative of two independent experiments. **(B)** C3b/iC3b on GFP transfected cells with or without M-Sez6L2 or H-DAF at 15% serum. ANOVA (P = 0.0016; F(2,6)=22.51). N = 3; one experiment with three replicates (representative of 3+ independent experiments). **(C)** Schematic of Sez6L2, Sez6, and Sez6L protein domain structures. **(D–I)** CHO cells were transfected with the indicated Myc-tagged cDNAs and processed as outlined in A with 15% C5 depleted serum, except that an anti-Myc antibody was used in place of GFP to identify transfected and expressing CHO cells. **(D)** 5% Contour plots of C3b/iC3b versus Myc fluorescence (top layer) and C3b/iC3b fluorescence histograms (bottom layer) of the same samples normalized to mode and compared to baseline cells not exposed to serum. For Contour plots, boxed regions highlight cells designated as Myc-positive (top box) and Myc-negative (lower box) populations. For C3b/iC3b histograms, dark grey, solid line population = Myc-positive cells; Light grey, dotted line population= Myc-negative cells; White, dashed grey line population = baseline. Representative of 4+ independent experiments. **(E)** Quantification of the average median C3b/iC3b fluorescence intensity from Myc-positive and Myc-negative cells within each sample. Statistics = t-tests. N = 3 (one experiment with three replicates; Representative of 4+ independent experiments). **(F)** Average median C3b/iC3b fluorescence intensities after normalization to the Myc-negative cells from each experimental group. ANOVA between Myc-positive cell populations (p<0.001; F(4, 15)=64.53). Sez6L2 inhibits C3b/iC3b opsonization at a level comparable to positive control MCP. Sez6 is a stronger complement inhibitor than Sez6L2 and Sez6L is a weaker inhibitor. **(F)** Average median Myc fluorescence intensity from Myc-positive cells. ANOVA (p<0.001; F(4, 15)=36.79). **(G)** Average % of Myc-positive cells in each experimental group (ANOVA, p=0.115; F(4, 15)=2.224). For sections **(F–H)**, N = 4 (four independent experiments). **(I)** Sez6 blocks complement opsonization more efficiently than Sez6L2 and Sez6L even when comparing similar levels of Myc surface expression. Average C3b/iC3b median fluorescence intensity normalized to internal Myc-negative populations for M-Sez6, M-Sez6L2, and M-Sez6L samples shown relative to the Myc median fluorescence intensity. N = 3 (one experiment with three replicates, Representative of three independent experiments). For all graphs *p < 0.05; **p < 0.01; ^#^p < 0.001 for all Myc-positive groups compared to M-CR2.”

The corrected caption is “Full Length Sez6L2 and Sez6L inhibit C3b/iC3b opsonization of CHO cells by the classical pathway. **(A, B)** Sez6L2 inhibits C3b/iC3b opsonization at a range of serum concentrations. CHO cells were transfected with plasmids for GFP alone or with Myc-tagged Sez6L2 (M-Sez6L2) or His-tagged DAF (H-DAF). CHO cells were coated with antibodies and exposed to 0-20% C5-depleted human serum for one hour and then immuno-stained with anti-C3b/iC3b antibodies and analyzed by flow cytometry. One experiment is shown that is representative of two independent experiments. **(B)** C3b/iC3b on GFP transfected cells with or without M-Sez6L2 or H-DAF at 15% serum. ANOVA (P = 0.0016; F(2,6)=22.51). N = 3; one experiment with three replicates (representative of 3+ independent experiments). **(C)** Schematic of Sez6L2, Sez6, and Sez6L protein domain structures. **(D–I)** CHO cells were transfected with the indicated Myc-tagged cDNAs and processed as outlined in A with 15% C5 depleted serum, except that an anti-Myc antibody was used in place of GFP to identify transfected and expressing CHO cells. **(D)** Contour plots of C3b/iC3b versus Myc fluorescence (top layer) and C3b/iC3b fluorescence histograms (bottom layer) of the same samples normalized to mode and compared to baseline cells not exposed to serum. For Contour plots, boxed regions highlight cells designated as Myc-positive (top box) and Myc-negative (lower box) populations. For C3b/iC3b histograms, dark grey, solid line population = Myc-positive cells; Light grey, dotted line population= Myc-negative cells; White, dashed grey line population = baseline. Representative of 4+ independent experiments. **(E)** Quantification of the average median C3b/iC3b fluorescence intensity from Myc-positive and Myc-negative cells within each sample. Statistics = t-tests. N = 3 (one experiment with three replicates; Representative of 4+ independent experiments). **(F)** Average median C3b/iC3b fluorescence intensities after normalization to the Myc-negative cells from each experimental group. ANOVA between Myc-positive cell populations (p<0.001; F(5, 18)=58.52). Sez6L2 inhibits C3b/iC3b opsonization at a level comparable to positive control MCP. Sez6 has no inhibitor activity and Sez6L is a weaker inhibitor. **(G)** Average median Myc fluorescence intensity from Myc-positive cells. Brown-Forsythe ANOVA (p<0.001; F*(5, 7.474)=15.26). **(H)** Average % of Myc-positive cells in each experimental group (ANOVA, p=0.354; F(4, 15)=1.192). For sections **(F–H)**, N = 4 (four independent experiments). **(I)** Sez6L2 blocks complement opsonization more efficiently than Sez6L and Sez6 even when comparing similar levels of Myc surface expression. Average C3b/iC3b median fluorescence intensity normalized to internal Myc-negative populations for M-Sez6, M-Sez6L2, and M-Sez6L samples shown relative to the Myc negative median fluorescence intensity. N = 3 (one experiment with three replicates, Representative of three independent experiments). For all graphs *p < 0.05; **p < 0.01; ^#^p < 0.001 for all Myc-positive groups compared to M-CR2.”

There was a mistake in the caption of **Figure 3** as published. The original caption stated

“Full Length Sez6L2, Sez6, and Sez6L inhibit C3b/iC3b opsonization of CHO cells by the alternative pathway. CHO cells were transfected with the indicated Myc-tagged cDNAs and then coated with a low level of antibodies and exposed to 20% C5-depleted human serum for one hour in the presence of 10 mM EGTA and 10 mM MgCl_2_ to block the classical pathway. Cells were then labeled with anti-C3b/iC3b and anti-Myc antibodies and analyzed by flow cytometry. **(A)** 5% Contour plots of C3b/iC3b versus Myc fluorescence (top layer) and C3b/iC3b fluorescence histograms (bottom layer) of the same samples normalized to mode and compared to baseline cells not exposed to serum. For Contour plots, boxed regions highlight cells designated as Myc-positive (top box) and Myc-negative (lower box) populations. For C3b/iC3b histograms, dark grey, solid line population = Myc-positive cells; Light grey, dotted line population= Myc-negative cells; White, dashed grey line population = baseline. Representative of 3+ independent experiments with technical replicates. **(B)** Quantification of the average median C3b/iC3b fluorescence intensity from Myc-positive and Myc-negative cells within each sample. N = 3 (one experiment with three replicates; Representative of 3+ independent experiments) Statistics = t-tests. E) Average median C3b/iC3b fluorescence intensities after normalization to the Myc-negative cells from each experimental group. ANOVA (p<0.001; F(4, 10)=74.47. N = 3 (3 independent experiments). For all graphs *p < 0.05; **p < 0.01 ^#^p < 0.001 for all Myc-positive groups compared to M-CR2.”

The corrected caption is “Full Length Sez6L2 and Sez6L inhibit C3b/iC3b opsonization of CHO cells by the alternative pathway. CHO cells were transfected with the indicated Myc-tagged cDNAs and then coated with a low level of antibodies and exposed to 20% C5-depleted human serum for one hour in the presence of 10 mM EGTA and 10 mM MgCl_2_ to block the classical pathway. Cells were then labeled with anti-C3b/iC3b and anti-Myc antibodies and analyzed by flow cytometry. **(A)** Contour plots of C3b/iC3b versus Myc fluorescence (top layer) and C3b/iC3b fluorescence histograms (bottom layer) of the same samples normalized to mode and compared to baseline cells not exposed to serum. For Contour plots, boxed regions highlight cells designated as Myc-positive (top box) and Myc-negative (lower box) populations. For C3b/iC3b histograms, dark grey, solid line population = Myc-positive cells; Light grey, dotted line population= Myc-negative cells; White, dashed grey line population = baseline. Representative of 3+ independent experiments with technical replicates. **(B)** Quantification of the average median C3b/iC3b fluorescence intensity from Myc-positive and Myc-negative cells within each sample. N = 3 (one experiment with three replicates; Representative of 3+ independent experiments) Statistics = t-tests. **C)** Average median C3b/iC3b fluorescence intensities after normalization to the Myc-negative cells from each experimental group. ANOVA (p<0.01; F(5, 11)=10.81. N = 2–3 independent experiments. For all graphs *p < 0.05; **p < 0.01 ^#^p < 0.001 for all Myc-positive groups compared to M-CR2”

In the abstract, there were mistakes regarding the Sez6 findings in sentences 5–9 that stated: “We show that Sez6 family members inhibit C3b/iC3b opsonization by the classical and alternative pathways with varying degrees of efficacy. For the classical pathway, Sez6 is a strong inhibitor, Sez6L2 is a moderate inhibitor, and Sez6L is a weak inhibitor. For the alternative pathway, the complement inhibitory activity of Sez6, Sez6L, and Sez6L2 all equaled or exceeded the activity of the known complement regulator MCP. Using Sez6L2 as the representative family member, we show that it specifically accelerates the dissociation of C3 convertases. Sez6L2 also functions as a cofactor for Factor I to facilitate the cleavage of C3b; however, Sez6L2 has no cofactor activity toward C4b. In summary, the Sez6 family are novel complement regulators that inhibit C3 convertases and promote C3b degradation.”

This has been corrected to read: “In this study, we demonstrate that Sez6L2 and Sez6L inhibit C3b/iC3b opsonization by the classical and alternative pathways with differing levels of efficacy, while Sez6 exhibits no complement inhibitory activity. Sez6L2 is a strong inhibitor, while Sez6L exhibits weaker activity comparable to that of the established complement regulator MCP. Using Sez6L2 as the representative family member, we show that it specifically accelerates the dissociation of C3 convertases. Sez6L2 also functions as a cofactor for Factor I to facilitate the cleavage of C3b; however, Sez6L2 has no cofactor activity toward C4b. In summary, Sez6L2 and Sez6L are novel complement regulators that inhibit C3 convertases and promote C3b degradation.”

The published article text contained multiple mistakes regarding conclusions about Sez6.

The original publication, **Introduction**, last sentence, stated: “We show here that members of the Sez6 family are novel complement regulators that inhibit the complement pathway at the level of C3 convertases.”

A correction has been made to the **Introduction**, last sentence, which now reads: “We show here that SEZ6L2 and SEZ6L are novel complement regulators that inhibit the complement pathway at the level of C3 convertases.”

The original publication’s **Result** section had a subsection title that read: “*Sez6 Proteins Limit C3b/iC3b Opsonization of CHO Cells by the Classical and Alternative Pathways*”.

A correction has been made to this results subsection title which now reads: “Sez6L2 and Sez6L Limit C3b/iC3b Opsonization of CHO Cells by the Classical and Alternative Pathways”

The original publication (results section, subsection: “Sez6 Proteins Limit C3b/iC3b Opsonization of CHO Cells by the Classical and Alternative Pathways”, paragraphs 2-4, starting with sentence 10 in paragraph 2) stated:

“M-Sez6L2, M-Sez6, M-Sez6L, and M-MCP all significantly decreased C3b/iC3b and M-CR2 significantly increased C3b/iC3b on expressing cells compared to non-transfected, Myc-negative cells within each experimental sample (**Figures 2D–F**). Overall, M-Sez6 was the most effective at decreasing C3b/iC3b opsonization as Myc-positive cells had only 17 ± 3% of the C3b/iC3b found on the Myc-negative cells in the same sample (p<0.001, Holm-Sidak MCT). M-Sez6L2 expressing cells had 52 ± 5% of C3b/iC3b compared to Myc-negative cells (p<0.001), M-Sez6L had 79 ± 3% (p=0.004), and M-MCP had 45 ± 6% (p<0.001). On the other hand, M-CR2 had 407 ± 78% the level of C3b/iC3b of internal Myc-negative cells P<0.001) (**Figure 2F**). These results show that Sez6 family members share the complement inhibitory function but have different levels of activity. Sez6 is the most effective inhibitor of the classical pathway. Sez6L2 is a moderate inhibitor and functions at a level comparable to MCP. Sez6L is a weak inhibitor.

“Because M-Sez6 expressed on the cell surface at twice the level of Sez6L2, Sez6L, and MCP (**Figure 2G**), but usually in a similar percentage of cells (**Figure 2H**), we wondered whether M-Sez6 was a more effective inhibitor simply because of the higher expression levels or whether there are intrinsic activity differences in the family members. Thus we gated the cell populations based on increasing levels of Myc surface expression to compare C3b/iC3b opsonization levels in cells with similar levels of surface M-Sez6, M-Sez6L or M-Sez6L2. The results suggest that all Sez6 family members inhibit complement better with higher surface expression. However, M-Sez6 was still more effective at limiting C3b/iC3b opsonization than M-Sez6L and M-Sez6L2 at all expression levels (**Figure 2I**).

“Next, full-length Sez6L2, Sez6, and Sez6L were analyzed for their ability to inhibit complement opsonization initiated primarily by the alternative pathway in the presence of Mg-EGTA. The results were similar to that obtained with the classical pathway and showed all Sez6 family members to be effective inhibitors of alternative pathway C3b/iC3b opsonization. However, their activities relative to each other were different (**Figures 3A–C**). M-Sez6L2 expressing cells had 34 ± 1%, M-Sez6 had 59 ± 2%, M-Sez6L had 52 ± 6%, M-MCP had 65 ± 2% and M-CR2 had 2624 ± 256% the level of deposited C3b/iC3b compared to Myc-negative cells within the same samples (**Figure 3C**). C3b/iC3b opsonization by the alternative pathway was less intense than the classical pathway and resulted in C3b/iC3b coated cells segregating into two main population peaks. The primary peak population had low C3b/iC3b opsonization. The second peak population contained less cells than the primary peak, but these cells were opsonized with high levels of C3b/iC3b that were almost equivalent to the levels found in the classical pathway assays. M-Sez6L2, M-Sez6, Sez6L, and M-MCP all prevented expressing cells from reaching the complement opsonization levels of the high C3b/iC3b peak population (**Figure 3A**). Alternatively, M-CR2 increased C3b/iC3b levels on expressing cells, shifting all Myc-positive cells into the high C3b/iC3b opsonized population. Interestingly, M-Sez6L2 expressing cells also had decreased C3b/iC3b levels in the lower peak population relative to the non-transfected, Myc-negative cells within the same sample (**Figures 3A, B**). However, these Myc-negative cells in M-Sez6L2 samples often had increased C3b/iC3b opsonization compared with the Myc-negative cells in other samples raising the question of whether M-Sez6L2 protects expressing cells at the expense of promoting complement opsonization on non-expressing cells. Nevertheless, the cells expressing Sez6 family members had less complement opsonization by the alternative pathway than non-expressing cells and their complement inhibitory activity equaled or exceeded the activity of the known complement regulator MCP. In summary, Sez6 family members are inhibitors of C3b/iC3b complement opsonization by both the classical and alternative pathways, but individual Sez6 family members vary in the efficacy of their complement inhibitory activity toward each pathway.”

A correction has been made to these paragraphs (results section, subsection: “Sez6 Proteins Limit C3b/iC3b Opsonization of CHO Cells by the Classical and Alternative Pathways”, paragraphs 2-4, starting with sentence 10 in paragraph 2) so they now read:

“M-Sez6L2, M-Sez6L, and M-MCP all significantly decreased C3b/iC3b while M-Sez6 exhibited no change. As expected, M-CR2 significantly increased C3b/iC3b on expressing cells compared to non-transfected, Myc-negative cells within each experimental sample (**Figure 2D–F**). M-Sez6L2 was the most effective at decreasing C3b/iC3b opsonization as Myc-positive cells had only 52 ± 5% of the C3b/iC3b found on the Myc-negative cells in the same sample (p<0.001, Holm-Sidak MCT). M-Sez6L had 79 ± 3% (p=0.011) and M-MCP had 45 ± 6% (p<0.001). On the other hand, M-CR2 had 407 ± 78% the level of C3b/iC3b of internal Myc-negative cells (P<0.001) (**Figure 2F**). These results show that Sez6L2 and Sez6L share the complement inhibitory function of the classical pathway but have different levels of activity.

“Because M-Sez6L2 expressed on the cell surface at a higher level than Sez6L (**Figure 2G**), and in a similar (or increased) percentage of cells (**Figure 2H**), we wondered whether M-Sez6L2 was a more effective inhibitor simply because of the higher expression levels or whether there are intrinsic activity differences in the family members. Thus, we gated the cell populations based on increasing levels of Myc surface expression to compare C3b/iC3b opsonization levels in cells with similar levels of surface M-Sez6, M-Sez6L or M-Sez6L2. The results demonstrate that both Sez6L2 and Sez6L inhibit complement better with higher surface expression. However, M-Sez6L2 was still more effective at limiting C3b/iC3b opsonization than M-Sez6L at all expression levels (**Figure 2I**). Meanwhile, Sez6 demonstrated no activity at any expression level.

“Next, full-length Sez6L2, Sez6, and Sez6L were analyzed for their ability to inhibit complement opsonization initiated primarily by the alternative pathway in the presence of Mg-EGTA. The results were similar to those obtained with the classical pathway and showed Sez6L2 and Sez6L to be effective inhibitors of alternative pathway C3b/iC3b opsonization. However, their activities relative to each other were different (**Figures 3A–C**). M-Sez6L2 expressing cells had 34 ± 1%, M-Sez6 had 75 ± 25%, M-Sez6L had 52 ± 6%, M-MCP had 65 ± 2% and M-CR2 had 2624 ± 256% the level of deposited C3b/iC3b compared to Myc-negative cells within the same samples (**Figure 3C**). C3b/iC3b opsonization by the alternative pathway was less intense than the classical pathway and resulted in C3b/iC3b coated cells segregating into two main population peaks. The primary peak population had low C3b/iC3b opsonization. The second peak population contained less cells than the primary peak, but these cells were opsonized with high levels of C3b/iC3b that were almost equivalent to the levels found in the classical pathway assays. M-Sez6L2, Sez6L, and M-MCP all prevented expressing cells from reaching the complement opsonization levels of the high C3b/iC3b peak population (**Figure 3A**). Alternatively, M-CR2 increased C3b/iC3b levels on expressing cells, shifting all Myc-positive cells into the high C3b/iC3b opsonized population. Interestingly, M-Sez6L2 expressing cells also had decreased C3b/iC3b levels in the lower peak population relative to the non-transfected, Myc-negative cells within the same sample (**Figures 3A, B**). However, these Myc-negative cells in M-Sez6L2 samples often had increased C3b/iC3b opsonization compared with the Myc-negative cells in other samples raising the question of whether M-Sez6L2 protects expressing cells at the expense of promoting complement opsonization on non-expressing cells. Nevertheless, the cells expressing Sez6L2 and Sez6L had less complement opsonization by the alternative pathway than non-expressing cells and their complement inhibitory activity equaled or exceeded the activity of the known complement regulator MCP.”

The published article contained multiple mistakes in the discussion section regarding conclusions about Sez6.

In the original publication, the **Discussion** section, paragraph 1, sentences 1–5 stated: “We have shown that Sez6, Sez6L, and Sez6L2 are all novel complement regulators that inhibit C3b/iC3b opsonization by the classical and alternative pathways. Sez6 was the most effective inhibitor of the classical pathway and functioned at a level equivalent to H-DAF and better than MCP. It also provided strong protection against the alternative pathway. Sez6L2 was a moderate inhibitor for the classical pathway that performed at a level similar to MCP and Sez6L2 was perhaps the most protective family member against the alternative pathway. Sez6L was a weak inhibitor towards the classical pathway but also functioned similar to MCP against the alternative pathway.”

A correction has been made to the **Discussion** section, paragraph 1, sentences 1-5, which now states:

“We have shown that Sez6L2 and Sez6L, but not Sez6, are novel complement regulators that inhibit C3b/iC3b opsonization by the classical and alternative pathways. Sez6L2 was the most effective inhibitor of the classical pathway and functioned at a level between H-DAF and MCP. It also provided strong protection against the alternative pathway. Sez6L was a weak inhibitor towards the classical pathway but functioned similar to MCP against the alternative pathway.”

In the original publication the **Discussion** section, paragraph 2, sentences 6-8, stated: “The synaptic and dendritic localization of Sez6L2 puts it in an ideal location to protect synapses and dendrites from complement-dependent pruning during development and may be a mechanism by which Sez6 proteins modulate synapse numbers and dendritic morphology. However, Sez6 proteins could also modulate complement activation levels affecting neurogenesis, neuronal migration, or immune reactions to various insults. Perhaps complement dysregulation explains the genetic association of the Sez6 family with multiple neurodevelopmental and psychiatric disorders including: autism, schizophrenia, intellectual disability, epilepsy, and bipolar disorder (**1**–**9**).”

A correction has been made to the discussion section, paragraph 2, sentences 6-8, which now reads: “The synaptic and dendritic localization of Sez6L2 puts it in an ideal location to protect synapses and dendrites from complement-dependent pruning during development and may be a mechanism to modulate synapse numbers and dendritic morphology. However, Sez6L2 and Sez6L could also modulate complement activation levels affecting neurogenesis, neuronal migration, or immune reactions to various insults. Perhaps complement dysregulation partially explains the genetic association of Sez6 family members with multiple neurodevelopmental and psychiatric disorders including: autism, schizophrenia, intellectual disability, epilepsy, and bipolar disorder (1–9).”

In the original publication, the **Discussion** section, paragraph 5, sentences 7-9, stated: “However, Sez6 and Sez6L do not have the correct five motif pattern; and yet, Sez6 was even more efficient at inhibiting C3b/iC3b opsonization by the classical pathway than Sez6L2. Interestingly, CSMD1, another brain expressed complement inhibitor associated with neurodevelopmental disorders, and perhaps a distant cousin of the Sez6 family, was also one of only two experimentally validated complement inhibitors that did not fit the five-motif pattern identified and reported by Ojha et al. (29, 80–83). As the core CCP motif pattern is somewhat different between Sez6L2 and Sez6, it is possible that there are unique features to their complement regulatory activity yet to be discovered.”

A correction has been made to the **Discussion** section, paragraph 5, sentences 7-9, so they now read: “However, Sez6 and Sez6L do not have the correct five motif pattern which could explain why they have no, or only weak, inhibitory activity. Interestingly, CSMD1, another brain expressed complement inhibitor associated with neurodevelopmental disorders, and perhaps a distant cousin of the Sez6 family, was also one of only two experimentally validated complement inhibitors that did not fit the five-motif pattern identified and reported by Ojha et al. (29, 80–83). As the core CCP motif pattern is somewhat different between Sez6L2 and Sez6L, it is possible that there are unique features to their complement regulatory activity yet to be discovered.”

In the original publication, the **Discussion** section, paragraph 6, sentences 6–7 stated: “The Sez6L variant we tested contained three CUB domains and was the least effective family member at blocking C3 deposition by the classical pathway. In contrast, the variants of Sez6 and Sez6L2 we tested had only two CUB domains and were much more effective at blocking the classical pathway.”

A correction has been made to the **Discussion** section, paragraph 6, sentences 6-7, which now read: “The Sez6L variant we tested contained three CUB domains and was less effective at blocking C3 deposition than the Sez6L2 variant we tested that had only two CUB domains.”

In the original publication, the **Discussion** section, paragraph 7, sentence 3 stated: “Increased expression of Sez6 family members has been linked to increased tumor growth and a poor prognosis in various cancers (17–23).”

A correction has been made to the **Discussion** section, paragraph 7, sentence 3, which now reads: “Increased expression of Sez6L2 and Sez6L has been linked to increased tumor growth and a poor prognosis in various cancers (17–23).”

In the original publication, the **Discussion** section, paragraph 7, last sentence, stated: “Testing for increased Sez6 family expression and employing strategies to block their complement inhibitory function alongside other therapeutic approaches may be necessary as it has been against tumors overexpressing other complement regulators like MCP, DAF, FH, or CD59.”

A correction has been made to the **Discussion** section, paragraph 7, last sentence, which now reads: “Testing for increased Sez6L2 or Sez6L expression and employing strategies to block their complement inhibitory function alongside other therapeutic approaches may be necessary as it has been against tumors overexpressing other complement regulators like MCP, DAF, FH, or CD59.”

The original version of this article has been updated.

